# Gene alterations at Drosophila inversion breakpoints provide *prima facie *evidence for natural selection as an explanation for rapid chromosomal evolution

**DOI:** 10.1186/1471-2164-13-53

**Published:** 2012-02-01

**Authors:** Yolanda Guillén, Alfredo Ruiz

**Affiliations:** 1 Departament de Genètica i de Microbiologia, Universitat Autònoma de Barcelona, 08193 Bellaterra (Barcelona), Spain

**Keywords:** Inversion breakpoints, mutation rate, chromosomal evolution, transposable elements, gene duplication, gene transposition, position effects

## Abstract

**Background:**

Chromosomal inversions have been pervasive during the evolution of the genus Drosophila, but there is significant variation between lineages in the rate of rearrangement fixation. *D. mojavensis*, an ecological specialist adapted to a cactophilic niche under extreme desert conditions, is a chromosomally derived species with ten fixed inversions, five of them not present in any other species.

**Results:**

In order to explore the causes of the rapid chromosomal evolution in *D. mojavensis*, we identified and characterized all breakpoints of seven inversions fixed in chromosome 2, the most dynamic one. One of the inversions presents unequivocal evidence for its generation by ectopic recombination between transposon copies and another two harbor inverted duplications of non-repetitive DNA at the two breakpoints and were likely generated by staggered single-strand breaks and repair by non-homologous end joining. Four out of 14 breakpoints lay in the intergenic region between preexisting duplicated genes, suggesting an adaptive advantage of separating previously tightly linked duplicates. Four out of 14 breakpoints are associated with transposed genes, suggesting these breakpoints are fragile regions. Finally two inversions contain novel genes at their breakpoints and another three show alterations of genes at breakpoints with potential adaptive significance.

**Conclusions:**

*D. mojavensis *chromosomal inversions were generated by multiple mechanisms, an observation that does not provide support for increased mutation rate as explanation for rapid chromosomal evolution. On the other hand, we have found a number of gene alterations at the breakpoints with putative adaptive consequences that directly point to natural selection as the cause of *D. mojavensis *rapid chromosomal evolution.

## Background

Chromosomal inversions are a common feature of genome evolution in many groups of animals and may play a significant role in adaptation, speciation and sex chromosome evolution [1-4]. The rate of rearrangement fixation varies significantly within and between animal groups [2,5]. The genus Drosophila shows one of the highest rates in all eukaryotes [6-8] at least partially because special cytological mechanisms in Diptera allow heterozygotes for paracentric inversions to circumvent the production of aneuploid gametes [1]. A striking extent of variation in rearrangement rate has been reported among different Drosophila lineages [6,9-12]. For instance, the fixation rate of inversions is higher in the Sophophora subgenus than in the Drosophila subgenus [10]. Also particular lineages such as *D. miranda *or *D. yakuba *exhibit an unusually rapid rate of chromosomal evolution [9,11]. Four factors may contribute to the variation among lineages in the rate of chromosomal rearrangement: generation time, population size, mutation rate and fitness effects of rearrangements. However, the actual reason for such variation is unclear and different studies invoke different explanations [9-12].

Chromosomal inversions can be generated by two major mechanisms. The first of them is ectopic recombination (or non-allelic homologous recombination, NAHR) between transposable elements (TEs) [13-15], segmental duplications [16,17] or short repeat sequences [18]. When ectopic recombination occurs between two copies of a TE inserted in opposite orientation at two different chromosomal sites, the resulting inverted chromosomal segment will be flanked by two chimeric TE copies bounded by exchanged target site duplications (TSD) [14,15]. The second mechanism is chromosomal breakage and erroneous repair of the free ends by non-homologous end-joining (NHEJ) [19]. Breakages can be simple double-strand breaks (DSB) or staggered single-strand breaks (SSB). In the second case, the consequence is the generation of inverted duplications at both sides of the inverted segment [11,20]. Thus, inversions generated in this way can be recognized by duplicated DNA segments (originally single-copy) in inverted orientation flanking the inverted chromosomal segment. The relative contribution of the two mechanisms to the generation of natural Drosophila inversions is not yet clear. In Dipterans, clear-cut evidence for the implication of TEs in their generation has been found for a few polymorphic inversions [15,21-23] but has never been found for fixed inversions [6,11,24,25]. On the other hand, breakage and repair by NHEJ may be the prevalent mechanism in *D. melanogaster *and its close relatives [11].

Several explanations have been put forward for the spread of inversions in populations [3]. Although in principle inversions could be neutral or underdominant and spread by genetic drift, this is probably unusual in Drosophila species given their elevated effective population size, of the order of 10^6 ^[26,27]. The traditional explanation for the adaptive significance of inversions is based on their recombination-reducing effect [28] that keeps together alleles at loci with epistatic effects on fitness, the "coadaptation" hypothesis [29]. An alternative model proposes that inversions capture a set of locally adapted genes and protect them from recombination with immigrant chromosomes [4,30]. Finally, inversions may spread in populations due to the direct mutational effects associated with their breakpoints, the "position effect" hypothesis [31]. This latter hypothesis has received so far little attention [32] but the relatively high gene density and compact structure of Drosophila genome (> 90% of euchromatin has functional annotations) [33,34] make position effects most likely. Available genomic sequences [35] provide the opportunity to investigate the structure of inversion breakpoints and ascertain their functional consequences.

*Drosophila mojavensis *has been an excellent model for the study of the genetics of ecological adaptation and speciation for more than fifty years [36-38] and it is now a useful model for genomic studies as the complete genome sequence is available [35,39]. *D. mojavensis *is a cactophilic species in the *repleta *group endemic to the deserts of the Southwestern USA and Northwestern Mexico, chiefly the Sonoran Desert (Arizona, Baja California and Sonora) the Mojave Desert and Santa Catalina Island in southern California. Natural populations are genetically differentiated and use different primary host plants, *Stenocereus gummosus *(pitaya agria) in Baja California, *Stenocereus thurberi *(organ pipe) in Arizona and Sonora, *Ferocactus cylindraceous *(California barrel) in Southern California and Opuntia spp. on Santa Catalina Island [40-42]. The ecological conditions of the Sonoran Desert are extreme (dry, arid and hot according to Köppen classification [43]) as attested by the fact that only four Drosophila species are endemic [41]. Accordingly, *D. mojavensis *is unusually thermotolerant and desiccation resistant [44-47]. In addition, *D. mojavensis *is the exclusive inhabitant of its chief host plants over most of its distribution range, in part because they contain large amounts of unusual lipids and triterpene glycosides that make them unsuitable for other Drosophila species [48,49].

**Figure 1:**
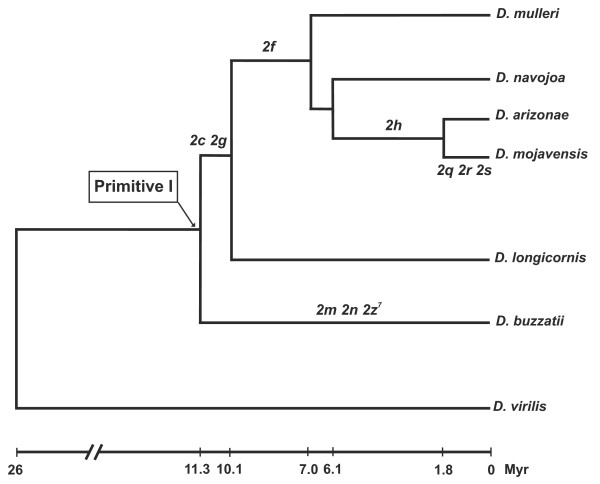
**Phylogenetic relationships and divergence times for seven species of the Drosophila subgenus**. Six species (*D. buzzatii, D. longicornis, D. mojavensis, D. arizonae, D. navojoa *and *D. mulleri*) belong to the *repleta *species group and the chromosome 2 inversions fixed in the *D. mojavensis *and *D. buzzatii *lineages are indicated [51,52]. Primitive I is the most recent common ancestor of *D. mojavensis *and *D. buzzatii *[52]. *D. virilis *is the outgroup species and belongs to the *virilis *species group. Phylogenetic relationships and divergence times are taken from [38, D.C.S.G. Oliveira, F.C. Almeida, P. O'Grady, W.J. Etges, M.A. Armella and R. DeSalle, personal communication].

The salivary gland chromosomes of *D. mojavensis *and its close relatives *D. arizonae *and *D. navojoa *were cytologically analyzed and the *D. mojavensis *standard chromosomal arrangement seemingly contain ten fixed inversions compared to Primitive I (the ancestor of the *repleta *group), one in chromosome X (*Xe*), seven in chromosome 2 (*2c*, 2*f*, 2*g*, 2*h*, 2*q*, 2*r *and 2*s*) and two on chromosome 3 (*3a *and *3d*) [50,51]. Five inversions (*3d, Xe, 2q, 2r *and *2s*) are exclusive to *D. mojavensis *whereas the rest are shared by other cactophilic Drosophila of the *mulleri *complex (see Figure 1). Thus, *D. mojavensis *is a chromosomally derived species that contains the highest number of fixed inversions in the entire *mulleri *complex [52]. Only one of *D. mojavensis *inversions (Xe) has been previously characterized at the molecular level [53]. Here we characterize all inversions fixed in *D. mojavensis *chromosome 2, the most dynamic of the five major chromosomes, and explore the causes of its rapid chromosomal evolution. Using comparative mapping of BAC-end sequences from *D. buzzatii *onto the *D. mojavensis *genome (see Figure 1), we identify the breakpoint regions of all inversions. We then annotate them by comparison with the genome of *D. virilis*, the closest relative with a sequenced genome [35] that represents the ancestral (non-inverted) arrangement. Our results provide information on the multiple causes that generated these inversions, reveal unreported associations of inversion breakpoints with duplicated and transposed genes, and shed light on the functional consequences of *D. mojavensis *inversions. Overall, our results suggest that rapid chromosomal evolution in *D. mojavensis *is not due to an increase in the rate of inversion generation but to its adaptation to the extremely harsh environment of the Sonoran Desert that was accompanied by strong natural selection.

## Results

### Identification of syntenic segments and breakpoint regions

We sequenced the ends of 1,152 *D. buzzatii *chromosome 2 BAC clones [54] and 1,870 BAC-end sequences (BES) mapped onto *D. mojavensis *chromosome 2 (see Methods for details). By comparing the chromosomal localization of the markers, we identified 20 syntenic segments (Additional file 1). *D. mojavensis *scaffold 6540, corresponding to chromosome 2 [55], is 34,148,556 bp long (coordinates begin at centromere). The most proximal marker in our map (segment 20) was located at position 1,721,255 bp whereas the most distal marker (segment 1) was located at position 34,039,404, i.e. only 109 kb from the end of the scaffold. The largest segment was number 15 with 5,926.5 kb and 426 markers whereas the smallest one was number 16 with 50.5 kb and 9 markers. The second-smallest segment was number 7 with 80.7 kb and 2 markers. This latter segment was exceptional as it was detected using comparative information from BAC clone 1B03 that has been fully sequenced [56]. In general, the markers were distributed homogeneously along the chromosome as indicated by the highly significant correlation (r^2 ^= 0.95, P < 0.001) between segment size and number of markers. The 20 syntenic segments amount to 30,830,590 bp, representing ~90.3% coverage of chromosome 2. The missing 3,317,966 bp are distributed between the endmost chromosomal regions (~5.3%) and the 19 breakpoint regions (~4.4%).

### Estimating the genomic distance

**Figure 2:**
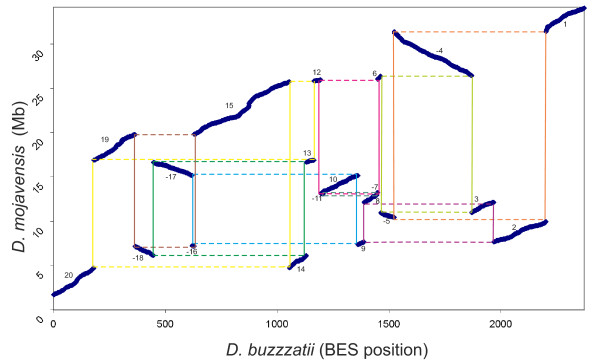
**Dot plot comparing the order of chromosome 2 markers between *D. mojavensis *and *D. buzzatii***. The x-axis represents the position of all markers in the physical map of *D. buzzatii *[54], while in the y-axis markers were ordered according to their coordinates in *D. mojavensis *scaffold 6540. Each syntenic segment is identified by a number with inverted segments indicated by a negative sign. Also shown is the breakpoint graph [57] generated by connecting consecutive syntenic segments along *D. buzzatii *chromosome 2 with continuous lines and those along the *D. mojavensis *chromosome 2 with dashed lines. The result is eight rectangles and a more complex cycle comprising two concatenated rectangles (yellow) that correspond to the 10 inversions fixed between the two species.

**Figure 3:**
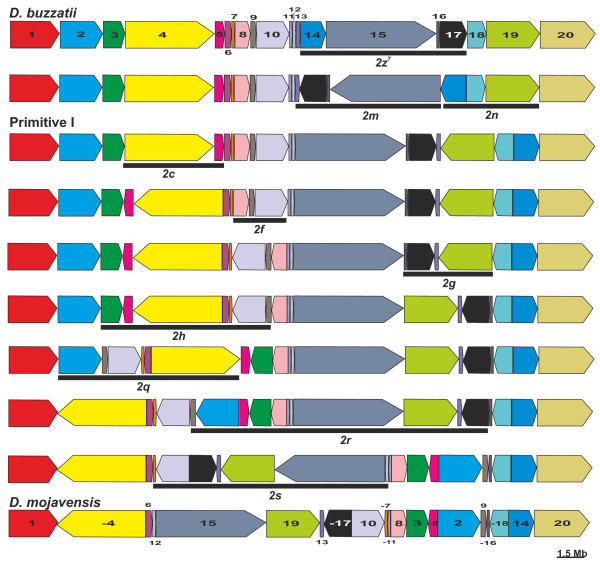
**Order and orientation of the 20 chromosome 2 conserved segments during the divergence between *D. buzzatii *and *D. mojavensis***. The genomic distance between the two species calculated using GRIMM software was 10 inversions. When inversions *2m, 2n *and *2z^7 ^*fixed in *D. buzzatii *[12,59] were subtracted, GRIMM software yielded a single scenario with seven inversions (*2c, 2f, 2g, 2h, 2q, 2r *and *2s*) fixed from Primitive I to *D. mojavensis*.

The order, size and orientation of the 20 conserved syntenic segments are shown in Figure 2. This breakpoint graph [57] contains nine cycles (represented with different colors), namely eight rectangles and a more complex cycle comprising two concatenated rectangles, suggesting that eight inversions and a more complex rearrangement are fixed in chromosome 2 since the divergence between *D. buzzatii *and *D. mojavensis*. GRIMM software [58] indicated that a minimum of 10 inversions are needed to transform the *D. buzzatii *chromosome 2 into that of *D. mojavensis *(Figure 3). Because there are 20 syntenic segments and 19 breakpoints, this implies one breakpoint reuse. Previous work in our laboratory [12] determined that three inversions, *2m, 2n *and *2z*^7^, have been fixed in chromosome 2 of *D. buzzatii *since its divergence from Primitive I, the most recent common ancestor with *D. mojavensis *(Figure 1). Furthermore, the breakpoints of these three inversions have been isolated and sequenced [59]. Inversions *2m *and *2n *are arranged in tandem and share the middle breakpoint. Thus we identified the complex cycle in the breakpoint graph (Figure 2) as corresponding to the *2mn *rearrangement and determined that seven inversions have been fixed in *D. mojavensis *since divergence from Primitive I. These seven inversions entail 14 breakpoints, i.e. they have independent breakpoints. GRIMM software [58] was run again to compare the arrangement of *D. mojavensis *chromosome 2 with that of Primitive I (inferred by subtraction of the three inversions fixed in *D. buzzatii*). The result was the single scenario shown in Figure 3.

In order to compare the inversions proposed by GRIMM with those detected previously using cytological methods (see Introduction), we located on the *D. buzzatii *physical map [54] those clones that mapped on each *D. mojavensis *breakpoint region and identified the chromosomal bands involved in each case. We corroborated the inversion breakpoints identified by cytogenetics and those detected by bioinformatics with an accuracy of three to five bands. Rearrangements detected cytologically and those proposed by GRIMM (Figure 3) did not only match in number but also the regions involved in each of them were in agreement, allowing for the differences between the precision of both techniques. However, three cytological breakpoint coincidences were not corroborated at the sequence level. The general agreement between cytogenetics and bioinformatics is remarkable because often these two approaches to chromosomal evolution seem to provide discordant results [60,61]. For instance, in Drosophila, comparative mapping has sometimes revealed fixed inversions overlooked by previous cytological studies [11,62,63].

### Delimitation and annotation of breakpoint regions

**Figure 4:**
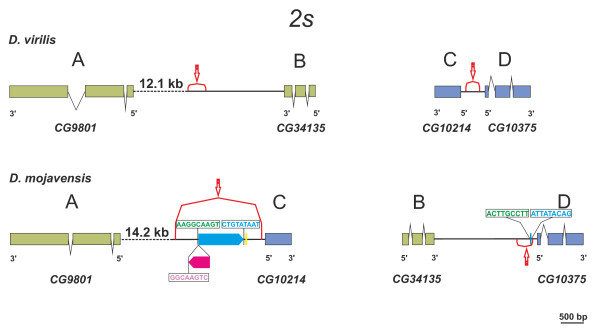
**Annotation of inversion *2s *distal and proximal breakpoint regions in *D. virilis *(non-inverted chromosome) and *D. mojavensis *(inverted chromosome)**. Genes are depicted as solid boxes (exons) linked by polygonal lines (introns) with the 5' and 3' ends showing the direction of transcription. Genes adjacent to the distal (AB) and proximal (CD) breakpoints of *D. virilis *are colored in green and blue, respectively. Orthologs in the distal (AC) and proximal (BD) *D. mojavensis *breakpoint regions are colored accordingly. Red curly brackets with an arrow indicate the breakpoint junctions. TE insertions are shown as solid rectangles: blue (*Bu*T5), purple (*Homo*3) or yellow (*Galileo*). Some TE insertions are flanked by TSDs insertions depicted in boxes above (or below) them. Dotted sections are not drawn to scale.

**Figure 5:**
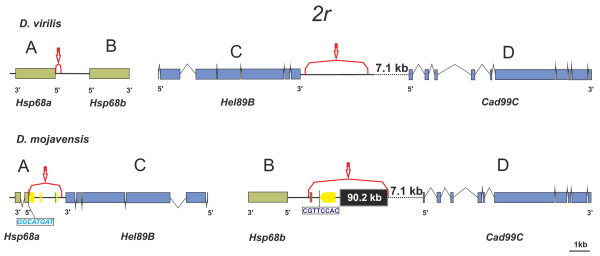
**Annotation of inversion *2r *distal and proximal breakpoint regions *in D. virilis *(non-inverted chromosome) and *D. mojavensis *(inverted chromosome)**. TE insertions shown as solid rectangles: yellow (*Galileo*), green (*Invader*) or brown (*Homo6*). The black box in the *D. mojavensis *BD region represents a 90.2 kb-block containing interspersed histone clusters and TEs. Other symbols as in Figure 4.

**Figure 6:**
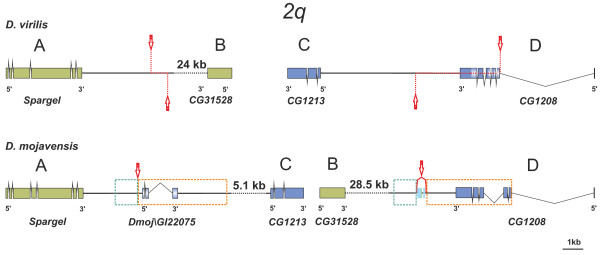
**Annotation of inversion *2q *distal and proximal breakpoint regions in *D. virilis *(non-inverted chromosome) and *D. mojavensis *(inverted chromosome)**. Inverted duplications in the *D. mojavensis *breakpoints are enclosed within dotted boxes, light blue (1 kb) or orange (4.3 kb). These duplications were presumably generated by staggered single-strand breaks in the parental chromosome represented by dotted red lines flanked by red arrows. *Dmoj\GI22075*, a novel gene not present in *D. virilis *breakpoint regions, was generated by the partial duplication of *CG1208*. Two unidentified TE insertions are found between the duplicated sequences in *D. mojavensis *BD region. Other symbols as in Figure 4.

Among the seven chromosome 2 inversions fixed in the *D. mojavensis *lineage, three (*2f, 2g *and *2c*) are shared between diverse species of the *mulleri *complex and must be between 7 and 11 myr old (Figure 1); another one (*2h*) is shared between *D. mojavensis *and *D. arizonae *only and should be between 2 and 6 myr old (Figure 1); the remaining three inversions (*2q, 2r *and *2s*) are exclusive of *D. mojavensis *and thus must be relatively young (less than 2 myr, Figure 1). We initially identified the 14 breakpoint regions of these seven inversions as those sequences between syntenic segments (Additional file 2). These regions varied between 9,776 bp and 480,695 bp. In order to narrow down the size of these regions, the corresponding sequences were blasted against the *D. virilis *genome (see Methods), which represents the parental (non-inverted) chromosome (Figure 1). We expect that breakpoint regions for each inversion will appear in *D. mojavensis *genome as AC (distal) and BD (proximal) but in *D. virilis *genome as AB (distal) and CD (proximal). Similarity comparisons of AC, BD, AB and CD sequences allowed us to reduce the size of the breakpoint regions to between 259 bp and 91,812 bp, on average 8.3% of the original breakpoint regions (Additional file 2). Five breakpoint regions were further reduced to about 71.1% of their previous size (on average) by excluding the coding sequences of orthologous genes. Once the new limits for the 14 breakpoint regions in *D. mojavensis *were established, we analyzed the similarity between the two breakpoint regions of each inversion using BLAST 2 sequences [64]. A summary list of the genes adjacent to the 14 inversion breakpoints is shown in Table 1 and a detailed annotation of the breakpoint regions of each inversion is shown in Figures 4, 5 and 6 for the most recent inversions (*2s, 2r *and *2q*) and Additional files 3, 4, 5 and 6 for the rest. TE content of all the breakpoint sequences (see Methods) is summarized in Additional file 7. Our analysis of the breakpoints provides significant information on the causes and consequences of the seven chromosome 2 inversions fixed in *D. mojavensis *(Table 1) that we present in the following sections.

**Table 1:** Chief features of inversion breakpoint regions in *D. mojavensis*

Inv	Breakpoints and adjacent protein-coding genes in *D. mojavensis*		TE copies at co-occurrent breakpoints	Inversion-associated inverted duplications	Preexisting duplications in parental genome	Transposition-associated genes and *D. virilis *lineage specific genes (underlined)	Gene gains (bold) and putative position effects
*2c*	AC	*Ligatin-GstD1a*					
			*BuT5*		*GstD1a-GstD1b*		*GstD1aGstD1b*
	BD	*Slbp-GstD1b*					

*2f*	AC	*αTub84B-Pli*					
						*Lsp1β*	
	BD	*CG1091-Lsp1β*					

*2g*	AC	*Dmoj\GI22722-CG4511*					
						*Dmoj\GI22722 CG32344* *CG2846 Dvir\GJ23779*	
	BD	*CG32344-spas*					

*2h*	AC	*pasha-ppk20*					
				7.1 kb from AB	*ppk20-ppk21*	*Dmoj\GI24456*	***Dmoj\GI23123***
	BD						

*2q*	AC	*Spargel-CG1213*					
				1 kb from AB 4.3 kb from CD	*CG1213-CG1208*		***Dmoj\GI22075***
	BD	*CG31528-CG1208*					

*2r*	AC	*Hsp68a-Hel89B*					
			*Galileo*		*Hsp68a-Hsp68b*	*Histone clusters*	*Hsp68a* *Hsp68b*
	BD	*Hsp68b-Cad99C*					

*2s*	AC	*CG9801-CG10214*					
				*BuT5*			*CG10375*
	BD	*CG34135-CG10375*					

### Generation of chromosomal inversions

In order to test for the implication of TEs in the generation of the seven inversions, we analyzed the TE content of the breakpoint regions and detected copies of a TE at both co-occurrent breakpoints in three inversions: *2s, 2r *and *2c *(Table 1). One of them, inversion *2s*, provides compelling evidence for the implication of the transposon *BuT5 *[65] in its generation. At the distal breakpoint, a 981-bp copy of *BuT5 *was found bounded by the 9-bp sequences AAGGCAAGT and CTGTATAAT (Figure 4). At the proximal breakpoint, we uncovered a 27-bp *BuT5 *fragment comprising 12 bp identical to one end and 15 bp identical to the other end, and thus resembling the footprints that transposons often leave behind at the donor site following excision [66,67] bounded by the 9-bp sequences ACTTGCCTT and ATTATACAG. These sequences can be interpreted as TSD produced at the time of the transposon insertion and its exchanged arrangement (ACTTGCCTT and CTGTATAAT are the inverted complementary versions of AAGGCAAGT and ATTATACAG, respectively) provides unequivocal evidence for the generation of inversion *2s *by ectopic recombination between *BuT5 *copies. Recently work in our laboratory has shown that an inversion fixed in *D. uniseta*, another related species that belongs to the *buzzatii *complex [56], has also been generated by ectopic recombination between *BuT5 *copies.

Two of our inversions provide evidence for generation by chromosomal breakage and erroneous repair by NHEJ [11,19,20] (Table 1). In the breakpoints of inversion *2h *we found evidence for a duplication of a 7.1-kb long segment containing three genes, *CG1792, Dmoj\GI23402 *and *pasha *(Additional file 3). When the two *D. mojavensis *breakpoint regions AC and BD were compared, we uncovered 10 blocks of similarity (E-value ≤ 9e-10) 45 to 641-bp long. These blocks are scattered in a 7,151-bp segment containing genes *CG1792, Dmoj\GI23402 *and *pasha *in the AC breakpoint, and within a 2,676-bp segment including *Dmoj\GI23123 *in the BD breakpoint (Additional file 3). This duplication can be explained by staggered SSB at the distal breakpoint in the parental chromosome. In the distal breakpoint of the derived chromosome (AC) the segment seems intact and the three genes fully functional whereas in the proximal breakpoint (BD), this segment has been reduced to 2.7 kb by several deletions (Additional file 3). Because the duplication was caused by the inversion, we estimated the age of the *2h *inversion using the divergence of those fragments non-coding for proteins and the Drosophila neutral substitution rate of 0.0111 [68] as 4.4 myr, which is in agreement with the phylogenetic distribution of inversion *2h *(Figure 1). In the breakpoints of inversion *2q *there are also inverted duplications. In this case, staggered SSB likely occurred at both breakpoints involving a ~1-kb segment at distal breakpoint (AB) and a 4.3-kb segment at proximal breakpoint (CD) (Figure 6). We estimated the age of inversion *2q *using the same procedure as before as 1.4 myr, a figure fully compatible with the phylogeny (Figure 1). There is a third case, where duplicated genes (*GstD1*) in opposite orientation are found at the two inversion *2c *breakpoints (Additional file 6). However, this observation is best interpreted as a breakage occurring at a preexisting duplication (see below).

### Preexisting gene duplications at breakpoints

We found four cases where inversion breakpoints fall between duplicated genes, i.e. there were preexisting gene duplications at the breakpoint regions (Table 1). In order to determine if this is in concordance with the random expectation, we estimated the number of intergenic regions localized between duplicated genes in *D. mojavensis *chromosome 2. Genes in this chromosome encode 3,407 out of the 14,595 *D. mojavensis *predicted proteins (23.34%). Thus there are 3,406 putative intergenic regions in this chromosome. According to the previous established criteria to consider two genes as duplicated copies (see Methods), we detected 215 intergenic regions between duplicated genes along the entire chromosome. We compared the two proportions by three different statistical methods. The χ^2 ^test with and without Yates correction (χ^2 ^= 11.526, P = 0.0007 and χ^2 ^= 8.111, P = 0.0044, respectively) indicated that 4/14 is significantly higher than the proportion expected at random (215/3406). Fisher exact test (P = 0.0098) corroborated this result. It could be argued that breakpoints are distributed at random in non-coding intergenic regions and that duplicated genes accumulate more breakpoints because their mean intergenic distance is longer than that between non-duplicated genes. We tested this possibility by calculating the intergenic distance for both duplicated and non-duplicated genes in *D. mojavensis *chromosome 2. A t-test showed that means are significantly different (t = 3.84, P = 0.0001), but the mean distance between duplicated genes is actually the shortest one. Thus, we conclude that there is an excess of breakpoints localized between duplicated genes with respect to the random expectation. Previously, another *D. mojavensis *inversion (*Xe*) was found to have a breakpoint adjacent to a gene duplication [53] and in primates, rearrangement breakpoints have been sometimes observed in the midst or adjacent to clustered gene families [69,70].

Two explanations can be put forward for these observations. Firstly, duplicated genes might cause instability and increased rate of DBS [71] or might be breakage "permissive" regions [70]. Alternatively, we suggest that the mobilization of a duplicated gene may entail in some cases beneficial position effects that might help the inversion to be fixed within the species. Two duplicated genes may have their evolution constrained because of shared regulatory sequences, their co-location in the same chromatin regulatory domain, or sequence homogenization by frequent conversion and ectopic recombination events. The re-location of one of the copies to a different chromosomal region might produce beneficial changes in the regulation of expression for one or the two copies and/or release them from evolutionary constraints (see below).

### Association of inversion breakpoints with gene transposition events

Gene content of chromosomal elements is generally conserved in the genus Drosophila although gene order has been scrambled extensively by fixed paracentric inversions [6]. However, there have been a number of genes that have been relocated between or within chromosomal elements by gene transposition or retroposition [72-74]. We searched the 28 genes adjacent to *D. mojavensis *inversion breakpoints in the 12 Drosophila genomes [35] for evidences of gene transposition and found that there are three genes involved in interchromosomal transposition events (*Lsp1beta, Dmoj\GI22722 *and *CG32344*) and another one (*Dmoj\GI24456*) is likely involved in a intrachromosomal transposition (Table 1). Furthermore, two genes are present in the *D. virilis *breakpoints regions but not in *D. mojavensis *and thus are likely also transposed genes. Finally, a large DNA block including several clusters of Histone genes have been inserted in the proximal breakpoint of inversion *2r *(Figure 5).

In order to test for an association of inversion breakpoints and gene transpositions, we first determined that 69 out of 514 interchromosomally relocated genes [73] are located in *D. mojavensis *chromosome 2. Then we compared our proportion of interchromosomal gene transpositions (3/28) to the general chromosome 2 proportion (69/3,407). The χ^2 ^test with and without Yates correction (χ^2 ^= 6.42, P = 0.0113; χ^2 ^= 10.22, P = 0.0014), and the Fisher exact test (P = 0.0199) indicated that 3/28 is significantly higher than the relation expected at random. This test is conservative as we did not take into account the putative intrachromosomal transposition of *Dmoj\GI24456*, the two *D. virilis *lineage specific genes or the 90-kb insertion at *2r *proximal breakpoint (see below). The association of inversion breakpoints and transposed genes is likely the result of the "fragility" or "permissivity" of these regions [8]. A clear example is the *2g *distal breakpoint region in *D. virilis *where three genes (*Dvir\GJ23449, Dvir\GJ23779 *and *CG32344*) have transposed to this region from different sources.

The *2r *proximal breakpoint (BD) harbors a big block of DNA (~90-kb) not found in any of the *D. virilis *breakpoints. This block contains at least five tandemly arranged copies of the Histone gene cluster [75,76]. The exact number of copies cannot be determined due to the presence of a ~10 kb sequence gap bounded by histone genes from different clusters. The block also comprises a large number of fragments annotated as repetitive sequences (110 ReAS elements that amount up to ~45% of the sequence). These elements tend to occur at regular intervals with a periodicity similar to that of the Histone gene clusters. In *D. melanogaster *the Histone complex (*HIS-C*) is located in chromosomal arm 2L (Muller element B) and comprises ~100 tandemly arranged copies of a cluster containing five Histone genes (*His1, His2B, His2A, His4 *and *His3*) [75,76]. Histone genes are often involved in transposition events. In the *repleta *group species, the ancestral and chief *HIS-C *(named *HIS-C1*) is likely located at chromosome 3, but there are other derived and probably smaller complexes (named *HIS-C2*) at chromosomes 3 and 4, implying at least two transposition events [62]. The insertion of a ~90-kb block containing several Histone gene clusters in the *2r *proximal breakpoint (BD) seems to represent yet another transposition event, which is probably specific to *D. mojavensis*. This block is not found in *D. virilis *in any of the two breakpoints and was not found in *D. mulleri *or *D. buzzatii *by *in situ *hybridization [62]. We suggest that the occurrence of this ~90-kb block is the result of the reintegration of an extrachromosomal circular DNA fragment (eccDNA) replicated by rolling circle replication [77] perhaps at the time of the inversion generation (when DSBs were available). This hypothesis explains the fact that this large insertion contains tandemly repeated coding (Histone) genes and TEs.

### Gene gains and changes in gene structure and/or expression

Two novel genes have been generated at the *D. mojavensis *breakpoints (Table 1). The gene *Dmoj\GI23123*, localized at *2h *proximal breakpoint (BD), comprises two exons encoding a 94-aa protein (Additional file 3). A similarity search indicated that it is related to the gene *pasha *(partner of *drosha, CG1800*), that is found in the distal breakpoint (AC). *pasha *has five exons and encodes a 655-aa protein with a double-strand RNA binding domain that is involved in primary miRNA processing, among other biological processes. Amino acid identity between *Dmoj\GI23123 *and *pasha *proteins is 93.5% over a 46-aa segment. Gene *Dmoj\GI23123 *has an unknown molecular function but a protein domain *PTHR13482 *involved in nucleic acid binding was detected with Interproscan [78]. In addition it is expressed according to modENCODE *D. mojavensis *DB http://www.modencode.org/, suggesting it is fully functional. This gene arose at the time of the inversion generation as a consequence of the duplication of a 7.1-kb segment originally containing three genes: *CG1792, Dvir\GJ23094 *and *pasha *(Additional file 3). Seemingly the duplicated copies of *CG1792 and Dvir\GJ23094 *were partially lost by deletion whereas the duplicated copy of *pasha *evolved into the novel gene *Dmoj\GI23123*.

Another novel gene, *Dmoj\GI22075*, is found at the distal breakpoint (AC) of inversion *2q *(Figure 6). It arose when this inversion was generated as a consequence of the duplication of a 4.3-kb segment containing a fragment of gene *CG1208*. This gene encodes a 508-aa protein that has glucose transmembrane transporter activity. *Dmoj\GI22075 *comprises three exons encoding a 153-aa protein with a 75-aa Major Facilitator Superfamily (MFS) domain [79]. The conservation of this domain indicates that it is a new functional gene and suggests that it has retained a MFS function.

Three inversions entail putative changes in gene structure and/or expression. Two *GstD1 *genes in opposite orientation were found at the two *D. mojavensis *breakpoints of inversion *2c *while only one is present in the proximal breakpoint (CD) of the *D. virilis *chromosome (Additional file 6). In order to ascertain the origin of these two genes, a phylogeny of *GstD *genes in *D. mojavensis *and *D. virilis *was built (Additional file 8). The two *Dmoj\GstD1 *genes are co-orthologs of the *Dvir\GstD1 *gene and we estimated the age of the duplication event that generated them (using divergence at synonymous sites) as 16 myr. Therefore, this duplication event took place before inversion *2c *and the inversion breakpoint occurred between two pre-existing duplicated *GstD1 *genes. *GstD1 *genes have been associated with the detoxification of insecticides as well as other chemical substances present at larval food sources [80]. Low et al. [81] detected that positive selection has operated on *GstD1 *and identified the parallel evolution of a radical glycine to lysine amino acid change (K171) in *D. melanogaster, D. pseudoobscura *and *D. mojavensis*. Matzkin [82] found additional evidence for the adaptive evolution of *Dmoj\GstD1a*, a gene that shows changes of expression level in response to the use of different host plants as larval substrates [83]. Inversion *2c *relocated *GstD1a *to a new chromosomal region and left the other copy *GstD1b *in the original position. This might have triggered changes in their gene expression regulation and/or evolutionary constraints. The two *D. mojavensis GstD1 *proteins differ by 14 aa including the critical 171 residue (where *GstD1a *has lysine but *GstD1b *has glutamic acid). In addition, according to *D. mojavensis *modENCODE DB the relocated *GstD1a *gene has seemingly a much higher expression level than the gene in the original location, *GstD1b*. We suggest that the *GstD1 *duplication and subsequent separation of the two copies by inversion *2c *may have had significant consequences for the adaptation of the lineage of *D. mojavensis *and related species of the *mulleri *complex to its cactophilic niche (Figure 1).

The *2r *distal breakpoint was localized in *D. virilis *between two *Hsp68 *genes oriented head-to-head (Figure 5). These two genes have the same structure and size (a single exon 1,935-bp long encoding a 644-aa protein) and nearly identical sequence (8 mismatches, 99.6% identity). However, in *D. mojavensis Hsp68a *(661 bp) is significantly shorter than *Hsp68b *(1,935 bp) and posses two exons encoding a 152-aa protein (Figure 5). The two genes only show conservation of a segment encoding 90-aa corresponding to a Heat Shock Protein domain (75% aa identity). We built a phylogeny of *Hsp68 *in 11 Drosophila genomes (*D. willistoni *is the only of the 12 species lacking *Hsp68*, Additional file 9). While a single *Hsp68 *gene is present in the six *melanogaster *group species, two copies oriented head-to-head are found in *D. pseudoobscura, D. persimilis, D. grimshawi *and *D. virilis*. Thus, this is likely to be the ancestral state. Nonetheless the phylogenetic tree shows a high similarity between the two *Hsp68 *copies present within each of these four species (Additional file 9) that can be interpreted as the result of concerted evolution by recurrent gene conversion or ectopic recombination [84]. In *D. mojavensis*, inversion *2r *relocated *Hsp68b *to a new chromosomal site along with its upstream regulatory sequences. A detailed sequence analysis confirms that the *Dmoj\Hsp68b *5' upstream region harbors two *cis*-regulatory motifs called HSEs (heat shock elements) modulating the expression of this gene [85]. But we also detect a third HSE, 683 bp upstream of the *Dmoj\Hsp68b *5' region, in opposite orientation to the previous two HSEs. This putative *cis-*regulatory motif is likely to correspond to the HSE of *Dmoj*\*Hsp68a*, apparently dragged by the inversion to the BD region upstream of *Dmoj\Hsp68b*. In addition, only ~2.5 kb upstream of this gene is the ~90-kb block of Histone genes and TEs (see above). Because TEs may influence chromatin organization [86] and this in turn is a significant determinant of gene expression [87,88], the insertion of this block is likely to have altered the expression level and/or pattern of *Dmoj\Hsp68b*. No promoter or regulatory HSE sequences were detected upstream of *Dmoj*\*Hsp68a *but according to *D. mojavensis *modENCODE DB this gene is being transcribed. It may be that it has recruited a new promoter (e.g. a fragment of the transposon *Galileo *located 3-bp from the initial codon; see Figure 5) and acquired a new function or it is on the way to becoming a pseudogene. It must be recalled that *Dmoj\Hsp68a *shows an altered structure and a high rate of sequence divergence (Additional file 8). In summary, we found that inversion *2r *has induced significant alterations of this gene in both structure and expression.

A footprint of a *BuT5 *was found in the *D. mojavensis *proximal breakpoint of inversion *2s*, 121 bp from the start codon of *CG10375 *(Figure 4). We used McPromoter http://tools.igsp.duke.edu/generegulation/McPromoter/[89] to look for the *Dmoj\CG10375 *promoter. A unique putative promoter region was located 115-bp 5' from the start codon. This putative promoter region (~100-bp) includes the *BuT5 *footprint and has a peak with high score (0.0505) located in region B (across the breakpoint). In addition it corresponds to a model 1 promoter (DNA replication related element). These observations contrast with the promoter region of *Dmel\CG10375 *that is model 3 (Motif6/Motif1) and has a narrow peak with a lower score (0.03925) and imply that the *2s *inversion and the *BuT5 *element have likely altered the expression of *Dmoj\CG10375*, presumably increasing it. Gene *CG10375 *has a single *DnaJ *domain and is the likely orthologous of human *DNAJC8 *gene, a member of the Hsp40 family.

## Discussion

In this study, we investigated the rapid chromosomal evolution of the *D. mojavensis *lineage that has fixed ten paracentric inversions since the *repleta *group ancestor, ~12 mya (Figure 1). Using *D. buzzatii *BAC-end sequences [54] and the genome sequences of *D. mojavensis *and *D. virilis *[35] we mapped, identified, annotated and analyzed all breakpoints of the seven inversions fixed in *D. mojavensis *chromosome 2, the most dynamic element. The results corroborated previous cytological analyses [51] and allowed us to provide significant information on the causes and consequences of these structural changes.

One hypothesis that may explain an accelerated chromosomal evolution rate is an increased mutation rate that generates more rearrangements per generation. This possibility was invoked to explain the high rate of chromosomal rearrangement between *D. miranda *and *D. pseudoobscura *[9]. Because inversions may be generated by TEs (see Introduction), one possible cause of high mutation rate is an increased transpositional activity. Therefore, it has been suggested that variation in transpositional activity of TEs might contribute to variation in rates of rearrangement fixation [12]. However, an increased mutation rate could also be due to the presence of other causes, both intrinsic and extrinsic (e.g. clastogenic chemicals or ionizing radiation). Overall, our results do not support this hypothesis because the inversions fixed in *D. mojavensis *seem the result of multiple generation mechanisms. We found direct evidence for the implication of transposon *BuT5 *in the generation of inversion *2s *and only circumstantial evidence for the implication of the transposons *BuT5 *and *Galileo *in inversions *2c *and *2r*, respectively. Inversions *2h *and *2q *harbor inverted duplications of non-repetitive DNA at the two breakpoints and were likely generated by staggered single-strand breaks and repair by non-homologous end joining. Finally, no definitive conclusion can be drawn about the generation of inversions *2f *and *2g*. It could be argued that the latter inversions might have been generated by TEs but subsequent changes in the breakpoint regions hindered our ability to find conclusive evidence for their implication. TE copies might have excised and move to other locations after generating the inversion (a hypothesis known as "hit-and-run" [24]), or be deleted due to the high rate of loss of nonfunctional DNA in Drosophila [90,91]. However, in the absence of supporting evidence we think that such inference is unwarranted.

In any case, the generation of inversion *2s *by transposon *BuT5 *is a significant finding because, in Dipterans, the implication of TEs in the generation of chromosomal inversions has been demonstrated for a few polymorphic rearrangements but never for fixed inversions (see Introduction). *BuT5 *is a MITE with unusual features [N. Rius, A. Delprat and A. Ruiz, personal communication]. It was discovered in *D. buzzatii *[65] but is present in the genome of most *repleta *group species, implying that it was probably already present in the ancestor ~16 mya [N. Rius, A. Delprat and A. Ruiz, personal communication]. In *D. mojavensis *is relatively abundant and transpositionally active but copy density in the dynamic chromosome 2 is not significantly higher than in the rest of chromosomes. These observations do not support the increased mutation hypothesis.

A second explanation for accelerated chromosomal evolution is an increase of the species' population size because the rate of fixation of selectively advantageous rearrangements is a direct function of population size [26]. The high rate of chromosomal evolution of the *D. yakuba *lineage in comparison with the *D. melanogaster *lineage was attributed to differences in population size [11]. The effective population size of *D. mojavensis *has been estimated as ~10^6 ^yet there is variation between populations in Baja California and Mainland Sonora [92,93]. However, there is no reason to assume that this is an unusually high figure. Population size of *D. arizonae*, its closest relative (Figure 1), is seemingly higher (or at least not lower) than that of *D. mojavensis *[92,93]. In contrast to *D. mojavensis*, which is fixed for five species-specific inversions, *D. arizonae *has only one [51]. Therefore, population size does not provide an adequate explanation for *D. mojavensis *rapid chromosomal evolution.

The third hypothesis is strong natural selection in a new environment that increases the number of fixed inversions. *D. mojavensis *is the only *mulleri *complex species inhabiting the Sonoran Desert. Other species of this complex, including its closest relatives *D. arizonae *and *D. navojoa *(Figure 1), live in less harsh environments of central and southern Mexico. Thus it must be presumed that adaptation to the extreme conditions of the Sonoran desert and to the exclusive host plants exploited by *D. mojavensis *must have required many adaptive genetic changes. Chromosomal inversions in Drosophila have been considered for decades as adaptive devices that spread in natural populations driven by natural selection (see Introduction). In fact there is ample evidence for the adaptive significance of polymorphic inversions (those that are segregating within species) but no such evidence has been provided for fixed inversions (those that appear as interspecific differences). We have found a variety of gene alterations at the breakpoints of *D. mojavensis *chromosome inversions and propose that these alterations contributed to their adaptive value. Overall, strong natural selection in a new harsh environment seems the most plausible cause for *D. mojavensis *rapid chromosomal evolution.

The alterations associated with the breakpoints of five *D. mojavensis *inversions include two gene gains (*Dmoj\GI23123 *and *Dmoj\GI22075*) and three putative alterations of gene structure and/or expression regulation (Table 1). We discuss these effects in turn. In *D. mojavensis *two new genes were generated associated to inversions *2q *and *2h*. As a consequence of the generation mechanism, staggered breakage and NHEJ repair, duplications of single-copy DNA were present at the breakpoints of these inversions at the onset. In the case of inversion *2q *this duplication included gene *CG1208 *(except for its first exon and upstream sequences, see Figure 6). The novel gene *Dmoj\GI22075 *is shorter than the original gene *CG1208 *but retains a MFS domain and could function as a sugar transmembrane transporter (if a new promoter has been recruited). In the case of inversion *2h *the duplicated segment included originally three genes (see Additional file 3). Only one gene (*Dmoj\GI23123*) seems to have survived. This gene is related to *pasha *(a gene involved in primary microRNA processing and gene silencing by miRNA) and according to modENCODE data, it is expressed. We suggest that novel genes might have contributed to the adaptive value of these inversions. Novel genes are widely recognized as a source of new functions [94] but inversion-associated duplication has not been considered a molecular mechanism that can generate new genes until very recently and only in prokaryotes [20].

The two most recent inversions, *2r *and 2s, that are exclusive to *D. mojavensis*, show putative alterations of structure and/or expression of heat shock protein (Hsp) genes. Hsp genes encode intra-cellular chaperones for other proteins and have been established as potential candidates for thermotolerance [95]. Hsp family harbors genes constitutively or inducibley expressed [96]. Heat-inducible genes are regulated by heat shock factor (HSF), which binds to HSE sequences [97] whereas other heat shock genes have an Hsf-independent regulation [98]. The distal breakpoint of inversion *2r *separated two previously linked and very similar Hsp68 genes (Figure 5). One of them, *Hsp68a*, remained in its original location but suffered a radical change in structure and sequence. It may have acquired a new function and expression pattern or may be in the process of becoming a pseudogene. The other gene, *Hsp68b*, apparently kept its HSE regulatory elements but was relocated to a completely new chromatin environment and is now found near a ~90-kb block composed of Histone genes and TEs. It is difficult to imagine that the expression of this gene has not been affected by these changes. Genes of the heat- inducible Hsp70 family (to which *Hsp68 *belongs) are positively related to thermotolerance but overexpression has survival costs and it seems that Hsp70 concentration has an intermediate optimum [44,99]. Some African populations of *D. melanogaster *with an exceptional thermotolerance show decreased levels of *Hsp70 *expression, caused by the insertion of TEs in one of the promoter regions of the *Hsp70Ba *gene [100]. In *D. mojavensis *an altered expression of Hsp68 genes could contribute to its exceptional thermotolerance. On the other hand, the proximal breakpoint of inversion *2s *was located upstream of *CG10375*, a gene with a DnaJ domain that likely belongs to the constitutively expressed Hsp40 family. In *D. melanogaster, hsp40 *is up-regulated in mutants lacking HSF [98] and probably has an essential role in thermotolerance [101]. Thus the changes induced by inversion *2s *and *BuT5 *insertion in the promoter of *CG10375 *likely conferred an adaptive advantage to *D. mojavensis *by increasing its thermotolerance. It can be hypothesized that the alterations of the heat inducible *Hsp68 *genes caused by inversion *2r *and the putative positive effect on the expression level of the constitutive gene *CG10375 *caused by inversion *2s *were in some way related and jointly contributed to the *D. mojavensis *unusual thermotolerance. This hypothesis might explain the rapid and exclusive fixation of both inversions in the *D. mojavensis *lineage.

By no means do we imply that the alterations unveiled at the breakpoints are the only cause of the *D. mojavensis *inversion adaptive significance. Inversions are not simple point mutations but complex structural changes involving hundreds of loci that may suffer further mutations along their evolutionary trajectory. Therefore we consider that the multiple explanations for the adaptive spread of inversions (see Introduction) are not mutually exclusive alternatives. This means that different inversions may be successful for different reasons but also that a single inversion may increase in frequency for different reasons along its trajectory. For instance, an inversion could gain an initial drive because of the alterations it causes at the breakpoints and incorporate afterwards interacting mutations that led to coadaptation or that increase local adaptation that further propel the inversion towards fixation. The molecular explanations for the role of chromosomal inversions in adaptation and speciation are only beginning to be disentangled.

## Conclusions

The breakpoint characterization of seven inversions fixed in *D. mojavensis *has provided significant information on the causes and consequences of these rearrangements. Multiple generation mechanisms seem to have acted in this lineage, an observation that does not support a mutational explanation for *D. mojavensis *rapid chromosomal evolution. On the other hand, we have found a set of alterations at the inversion breakpoints with potential adaptive significance, including novel genes and changes in structure and/or expression of adjacent genes. Overall, our results are consistent with natural selection as an explanation for the rapid chromosomal evolution in this specialist organism living under extreme ecological conditions.

## Methods

In order to map and characterize the breakpoints of *D. mojavensis *chromosome 2 inversions we used a three-step approach: (1) End sequencing of a set of BAC clones from *D. buzzatii *chromosome 2; (2) Mapping of the resulting BAC-end sequences (BES) onto the *D. mojavensis *genome in order to determine the number and chromosomal span of the inversions fixed during the divergence of the two lineages; (3) Identification and annotation of the breakpoint regions using the *D. virilis *genome as representative of the parental (non-inverted) genome. Chromosome 2 of *D. mojavensis *differs by 42 chromosomal inversions from the homologous element in *D. virilis *[6]. The use of the *D. buzzatii *BES allowed us to identify and characterize those inversions fixed in the *D. mojavensis *lineage after its divergence from the *repleta *group ancestor (see Figure 1).

### BAC end sequencing

We selected 1,152 clones from the *D. buzzatii *BAC library homogenously distributed along the 28 contigs of the chromosome 2 physical map [54]. To minimize redundancy we choose overlapping clones but with different restriction patterns. This was done using the information provided by the fingerprinting analysis of BAC clones that is available at http://www.bcgsc.ca/platform/bioinfo/software/ice. The 1,152 clones were rearrayed into 96 well plates (CHORI, Children's Hospital Oakland Research Institute) and both ends of each clone were sequenced (Macrogen Inc., Seoul, Korea) using the universal T7 primer and the modified universal SP6 primer (ATTTAGGTGACACTATAGAAGG) for PCR amplifications at the forward and reverse ends, respectively. We generated 2,127 reads over 400 bp in length, a success rate of 92.32%. Length distribution of BAC-end sequences (BES) for the two primers were similar with a pronounced mode around 700-800 bp (Additional file 10). If only high-quality BES (Q≥20) are taken into account, 80.82% of all sequences had over 400 bp in length. Our goal was to maximize the number of clones with both ends sequenced (paired BES) to increase coverage and the chances to capture all inversion breakpoints. Thus, a total of 1,004 of the original 1,152 BAC clones (87.2%) produced paired BES, whereas 119 clones (10.3%) produced a single BES. All BES were filtered with *Geneious*^® ^software [102] using *VecScreen *database in order to identify and remove additional plasmidic sequences.

### Mapping *D. buzzatii *BES onto the *D. mojavensis *genome

All *D. buzzatii *BES were tested for similarity to the *D. mojavensis *genome by BLASTN. This multiple search was carried out with the parameter set '-e' 1e-20, '-W' 7, '-r' 2, '-q' 3, '-G' 5 and '-E' 2 (e-value, word size, reward for a nucleotide match, penalty for a nucleotide mismatch, gap opening cost and gap extension cost, respectively). The values of the rest of the parameters were assigned by default. A masked CAF1 version of *D. mojavensis *genome, which is available at *FlyBase *website ftp://ftp.flybase.net/genomes/aaa/transposable_elements/ReAS/v1/CAF1_masked/, was used as reference for these blast searches. The use of a masked genome based on the ReAS library [103] allowed us avoiding results with multiples hits due to repetitive sequences, such as TEs or heterochromatic fragments. Only those hits localized at chromosome 2, which is uniquely represented by scaffold 6540 [55], were considered. Of these, we only took into account the hits that had a minimum length of 50 bp (10% of sequence mean length, approximately). The rest of the hits were discarded, including multiple hits for different scaffolds (except BLAST outputs composed by multiple hits in scaffold 6540 only). All validated hits, i.e., those that met the above criteria, were reordered based on the coordinates of *D. mojavensis *genome.

From the initial 2,304 BES, 1,933 (83.9%) matched any region of *D. mojavensis *genome while 1,870 (81.2%) mapped onto chromosome 2 resulting in 2,421 hits (Additional file 10). The number of hits exceeds the number of BES because some BES yielded more than one hit. In most cases the hits produced by a single BES were concatenated, i.e. mapped at adjacent sites in the *D. mojavensis *genome.

We included in our study a number of BES generated in previous works [56,59] reaching a total of 2,456 hits. Assuming that chromosome 2 is ~34 Mb long [55], we estimated an average density of one hit or marker every 13.8 kb. The distributions of hit size, e-value and percent identity are shown in (Additional file 10). Hit size was over 400 bp in 50% of all cases, and we did not obtain hits with a length lower than 50 bp due to filtering restrictions. The distribution of e-value was similar for BES from both primers, T7 and SP6, and shows a prominent peak (18.32% of all hits) at an e-value equal to 0 (Additional file 10). Finally, the distribution of percent identity between the *D. buzzatii *BES and the *D. mojavensis *genome sequences showed a bell-shaped distribution with an average value of 83.1% (Additional file 10).

### A revised version of *D. buzzatii *physical map of chromosome 2

The published version of *D. buzzatii *physical map [54] comprises 28 contigs on chromosome 2. Another contig, 1031, has been anchored in chromosome 2 between contigs 1090 and 1181 in a recent mapping work [56]. Here, only four out of the 29 contigs, 1331, 987, 1330 and 1344, were not mapped to chromosome 2 and accordingly are likely to be misassembles or artifacts. We removed them from the revised version. The information provided by the comparative mapping of *D. buzzatii *BES onto the *D. mojavensis *genome allows us to assess the presence or absence of overlaps or gaps between contigs and estimate gap size. Supposing that there are no rearrangements or large indels involving contiguous sequences from adjacent contigs, we expect that contigs overlapping in *D. mojavensis *will also overlap in *D. buzzatii*, and vice versa. Based on this premise and assuming that *D. buzzatii *chromosome 2 has a similar size to that of *D. mojavensis*, we deduce that 15 of the putative gaps between contigs do not exist, i.e. we consider them closed gaps. In addition, we estimated the size of seven gaps between contigs of chromosome 2 as 20-240 kb. Finally, for the remaining two gaps, corresponding to breakpoint regions (see below), we estimated an upper bound for size. In summary, the new version of the map of *D. buzzatii *chromosome 2 comprises 10 contigs covering ~90% of chromosome 2 and contains 9 gaps that amount to ~5%. The remaining 5% correspond to the endmost (proximal and distal) regions that remain unmapped (see below).

### Identification of syntenic segments

Each hit in the *D. mojavensis *genome was associated with its corresponding clone in the *D. buzzatii *physical map [54]. In this way, we could infer the number, arrangement and orientation of the conserved segments between *D. buzzatii *and *D. mojavensis*. With a single exception (Additional file 1), no syntenic segments were accepted with less than nine hits. Only 77 markers (3%) were not part of any syntenic segment. We guess that these markers represent common elements scattered throughout the genome, such as structural or functional domains or regulatory sequences, or represent gene transposition events. Some BES did not map to any *D. mojavensis *genome region. This might be caused by incompletely sequenced reads (those which were few bp long), regions with high sequence divergence between *D. mojavensis *and *D. buzzatii *or repetitive fragments. Finally, the centromere was not included in any syntenic segment owing to the lack of markers in this region (a masked *D. mojavensis *genome was used as reference).

### Genomic distance

Once established the order and orientation of all syntenic segments in chromosome 2 between *D. buzzatii *and *D. mojavensis*, we estimated the genomic distance between the two species using GRIMM software [58]. The genomic distance is the minimum number of chromosomal rearrangements that differentiate two species [104]. The number of rearrangements estimated in this way was the sum of all inversions that had been fixed in the two lineages, *D. mojavensi*s and *D. buzzatii*, since their divergence from Primitive I [12]. The three inversions, *2m, 2n *and *2z*^7^, fixed in the *D. buzzatii *lineage have been previously identified [12] and their breakpoints characterized at the molecular level [58]. This allowed us to subtract them from the total and infer the inversions fixed in the other lineage, i.e. from Primitive I to *D. mojavensis*.

### Breakpoint analysis

We identified the breakpoint regions as the *D. mojavensis *genome sequences located between each pair of adjacent syntenic segments and estimated their size as the distance from the final marker in one syntenic segment to the initial marker in the next syntenic segment. The two breakpoints belonging to the same inversion were associated with the aid of GRIMM results. Once the two breakpoints of each inversion were identified, we proceeded to confirm these results by comparing the breakpoint regions sequences with *D. virilis *genome using FlyBase GBrowse http://flybase.org/cgi-bin/gbrowse/.

*D. virilis *is the phylogenetically closest species to *D. mojavensis *whose genome has been sequenced [35]. For this reason it was used as reference for the breakpoint comparative analysis. In order to narrow down the breakpoint regions, we blasted the breakpoint sequences against the *D. virilis *genome CAF1 masked version, also available at FlyBase website ftp://ftp.flybase.net/genomes/aaa/Transposable_ elements/ReAS/v1/CAF1_masked/. A threshold e-value of 1e-3 was set to take into account the phylogenetic distance between *D. virilis *and *D. mojavensis *(Figure 1). All the BLASTN searches were performed with the parameters '-W' 7, '-r' 2, '-q' 3, '-G' 5 and '-E' 2. All hits for each breakpoint sequence were ordered according to *D. mojavensis *coordinates and the coordinates defined by the similarity loss between *D. mojavensis *and *D. virilis *were the new breakpoint limits. A final refinement of the breakpoint regions was carried out comparing the structures of those genes adjacent to the breakpoints in *D. mojavensis *with their respective orthologs in *D. virilis *(annotations extracted from FlyFase http://flybase.org) [105]. If the exon number and gene size were the same or very similar in the two orthologs, coding sequences still present in the *D. mojavensis *breakpoint regions were excluded from them, confining breakpoints to the intergenic space.

To detect orthologs in the *D. virilis *genome we downloaded from FlyBase [105] all the nucleotide sequences corresponding to the pair of genes adjacent to each *D. mojavensis *breakpoint, and then we used them as queries for BLASTN searches against *D. virilis *genome. We considered as ortholog that gene whose sequence in *D. virilis *was covered by the most significant hit of that search. To ensure the results, each BLAST search was repeated by exchanging the reference genome with *D. mojavensi*s using as queries those *D. virilis *genes sequences putatively identified as orthologs in the first BLAST results (the *Reciprocal Best Hit *method, [106]). The function of genes adjacent to the breakpoints was inferred from the function of *D. melanogaster *orthologs in FlyBase and, for those genes without *D. melanogaster *orthologs, by searching for conserved domains using Interproscan [78] or NCBI Conserved Domain Database [107].

### Search of transposable elements

We generated a database with all the *D. mojavensis *breakpoint sequences. Then we identified all the TEs present at the breakpoint regions by a set of BLASTN searches against DPDB [108], non redundant nucleotide database [109] and RepBase update [110]. We also used RepeatMasker [111] to detect repeats and TEs. Finally, we performed a set of BLAST searches against the breakpoint database using as queries a group of known TEs: *Galileo *from *D. mojavensis *(BK006357.1) [112], *Newton-1, Newton-2, Kepler-1 and Kepler-5 *from *D. buzzatii *[113], and *BuT5 *from *D. mojavensis *[N. Rius, A. Delprat and A. Ruiz, personal communication].

### Detection of tandemly arranged duplicated genes

A number of the characterized inversion breakpoints were located between tandemly arranged duplicated genes in the parental (*D. virilis *genome). In order to test whether this number was expected under a random breakage model, we analyzed all the intergenic regions between duplicated genes in *D. mojavensis *chromosome 2. We first downloaded a database of predicted proteins for this species available at FlyBase website (version r1.3 of Feb 18, 2010). We extracted from this database all the proteins encoded by genes in scaffold 6540 (chromosome 2) and reordered them based on their gene position on this chromosome. Then, we carried out a search for pairs of similar proteins encoded by adjacent genes using BLAST 2 sequences (*bl2seq*) [64] with a cutoff *e-value *of 1e^-30^. Based on the characteristics of duplicated genes found at breakpoint regions we considered that a pair of proteins was encoded by duplicated genes when the sequence identity between them was over 33% and at least one of the hits was longer than 57% of the shortest query length. Finally we counted the number of intergenic regions located between duplicated genes according to *bl2seq *results.

## Authors' contributions

YG carried out the computational analysis. AR conceived and coordinated the study. YG and AR wrote the manuscript. All authors read and approved the final manuscript.

## Supplementary Material

Additional file 1 **Size, coverage and coordinates of syntenic segments between *D. mojavensis *and *D. buzzatii *chromosome 2**. Click here for file

Additional file 2 **Data for genome mapping of inversion breakpoint regions in the *D. mojavensis *genome**. Click here for file

Additional file 3 **Annotation of inversion *2h *breakpoint regions**. Annotation of inversion *2h *distal and proximal breakpoint regions in *D. virilis *(non-inverted chromosome) and *D. mojavensis *(inverted chromosome). Inverted duplications in the *D. mojavensis *breakpoints are enclosed within dotted boxes, orange color. That in region AC (7.1 kb) is intact whereas that in region BD (2.7 kb) has suffered several deletions. These duplications were presumably generated by staggered single-strand breaks in the parental chromosome represented by a dotted red lines flanked by red arrows. A fragment of *Bu*T3 is shown as a blue rectangle in region BD. Other symbols as in Figure 4. Click here for file

Additional file 4 **Annotation of inversion *2g *breakpoint regions**. Annotation of inversion *2g *distal and proximal breakpoint regions in *D. virilis *(non-inverted chromosome) and *D. mojavensis *(inverted chromosome). Two *D. virilis *lineage specific genes are shown as grey rectangles. Other symbols as in Figure 4. Click here for file

Additional file 5 **Annotation of inversion *2f *breakpoint regions**. Annotation of inversion *2f *distal and proximal breakpoint regions in *D. virilis *(non-inverted chromosome) and *D. mojavensis *(inverted chromosome). Symbols as in Figure 4. Click here for file

Additional file 6 **Annotation of inversion *2c *breakpoint regions**. Annotation of inversion *2c *distal and proximal breakpoint regions in *D. virilis *(non-inverted chromosome) and *D. mojavensis *(inverted chromosome). Phylogenetic analysis of *GstD *genes (Additional file 8) indicates that the *2c *inversion occurred after the duplication of the GstD1 gene in the parental chromosome. The *GstD9 *gene has lost its function in *D. mojavensis *becoming a pseudogene. Other symbols as in Figure 4. Click here for file

Additional file 7 **TE content of inversion breakpoint regions in *D. mojavensis***. Click here for file

Additional file 8 **Neighbor-Joining phylogenetic tree of GstD genes in *D mojavensis *and *D virilis***. Neighbor-Joining phylogenetic tree of GstD genes in D *mojavensis *and *D virilis*. Bootstrap values data for all tree nodes are shown. Phylogenetic analysis was conducted with MEGA4 [114]. Evolutionary distances were computed using the Maximum Composite Likelihood method. Click here for file

Additional file 9 **Neighbor-Joining phylogenetic tree of Hsp68 genes of 12 sequenced Drosophila species**. Neighbor-Joining phylogenetic tree of Hsp68 genes of 12 sequenced Drosophila species. *D. persimilis, D. pseudoobscura, D. grimshawi, D. virilis *and *D. mojavensis *have two copies of the Hsp68 gene, while *D. sechellia, D. simulans, D*. *melanogaster, D. erecta, D. yakuba *and *D. ananassae *only one. No Hsp68 gene has been detected in *D. willistoni*. Bootstrap values for all tree nodes are shown. Phylogenetic analysis was carried out using MEGA4 [114]. Evolutionary distances were computed using the Maximum Composite Likelihood method. Click here for file

Additional file 10 **Statistics of *D. buzzatii *BAC end sequences**. Description: Size distribution of *D. buzzatii *BAC end sequences (A) and distribution of size (B), E-value (C) and % identity (D) for hits generated blasting them against the *D. mojavensis *genome. See text for details. Click here for file
