# Supplementary material for: Gene alterations at Drosophila inversion breakpoints provide prima facie evidence for natural selection as an explanation for rapid chromosomal evolution
Source: BMC Genomics. 2012 Feb 1;13:53. doi: 10.1186/1471-2164-13-53 (PMC3355041; doi:10.1186/1471-2164-13-53)
Supplement: Additional file 1 — Size, coverage and coordinates of syntenic segments between D. mojavensis and D. buzzatii chromosome 2. [file 1471-2164-13-53-S1.PDF]

Additional file 1. Size, coverage and coordinates of syntenic segments between *D. mojavensis* and *D. buzzatii* chromosome 2.

Syntenic segment	Begin	End	Size (bp)	Coverage (number of markers)
20	1721255	4692600	2971346	183
14	4743675	6104645	1360971	75
18	6137184	7154445	1017262	82
16	7172282	7222783	50502	9
9	7365221	7654616	289396	28
2	7664393	9955684	2291292	233
5	10436380	10941168	504789	61
3	10957988	12125979	1167992	98
8	12137327	12970351	833025	57
11	13067258	13124282	57025	10
7	13151145	13231800	80656	2*
10	13381003	15145288	1764286	155
17	15167727	16621615	1453889	173
13	16659223	16888133	228911	34
19	16903388	19774789	2871402	184
15	19825375	25751837	5926463	426
12	25824411	25953117	128707	30
6	25968812	26375571	406760	13*
4	26441888	31225471	4783584	350
1	31397073	34039404	2642332	172

*The complete sequence of the clone 01B03 was used as a marker (Prada 2010). This sequence mapped in two different regions of the chromosome 2, one belonging to the syntenic segment 6 and the other to the syntenic segment 7.
