# Supplementary material for: Gene alterations at Drosophila inversion breakpoints provide prima facie evidence for natural selection as an explanation for rapid chromosomal evolution
Source: BMC Genomics. 2012 Feb 1;13:53. doi: 10.1186/1471-2164-13-53 (PMC3355041; doi:10.1186/1471-2164-13-53)
Supplement: Additional file 2 — Data for genome mapping of inversion breakpoint regions in the D. mojavensis genome. [file 1471-2164-13-53-S2.PDF]

Additional file 2. Genome mapping of inversion breakpoint regions in the *D. mojavensis* genome.

Inversion	BP	Neighboring syntenic segments	Initial BES mapping			Similarity to *D. virilis* genome			CDS of neighboring genes		
			D. mojavensis coordinates		BP region (bp)	*D. mojavensis* coordinates		BP region (bp)	*D. mojavensis* coordinates		BP region (bp)
			Begin	End		Begin	End		Begin	End	
2c	Distal	3 – 5	10941169	10957987	16819	10951558	10952204	647			
	Proximal	4 – 6	26375572	26441887	66316	26378790	26379233	444			
2f	Proximal	11 – 8	12970352	13067257	96906	13059356	13061415	2060	13060199	13061415	1217
	Distal	10 – 7	13231801	13381002	149202	13376979	13377791	813			
2g	Proximal	16 – 18	7154446	7172281	17836	7159934	7161052	1119			
	Distal	15 -19	19774790	19825374	50585	19804465	19805612	1148	19804465	19805311	847
2h	Distal	2 – 9	7654617	7664392	9776	7664068	7664784	717	7664342	7664784	443
	Proximal	8 – 3	12125980	12137326	11348	12128366	12129507	1142	12128366	12129293	928
2q	Proximal	5 – 2	9955685	10436379	480695	10420224	10422204	1981			
	Distal	1 – 4	31225472	31397072	171601	31254883	31255399	517			
2r	Proximal	9 – 16	7222784	7365220	142437	7230145	7321956	91812			
	Distal	17 – 10	15145289	15167726	22438	15160462	15162581	2120	15160909	15162581	1673
2s	Proximal	7 – 11	13124283	13151144	26862	13149238	13149496	259			
	Distal	6 – 12	25953118	25968811	15694	25966954	25968814	1861			
