# Supplementary figures and images for: Gene alterations at Drosophila inversion breakpoints provide prima facie evidence for natural selection as an explanation for rapid chromosomal evolution
Source: BMC Genomics. 2012 Feb 1;13:53. doi: 10.1186/1471-2164-13-53 (PMC3355041; doi:10.1186/1471-2164-13-53)

2h

*D. virilis*

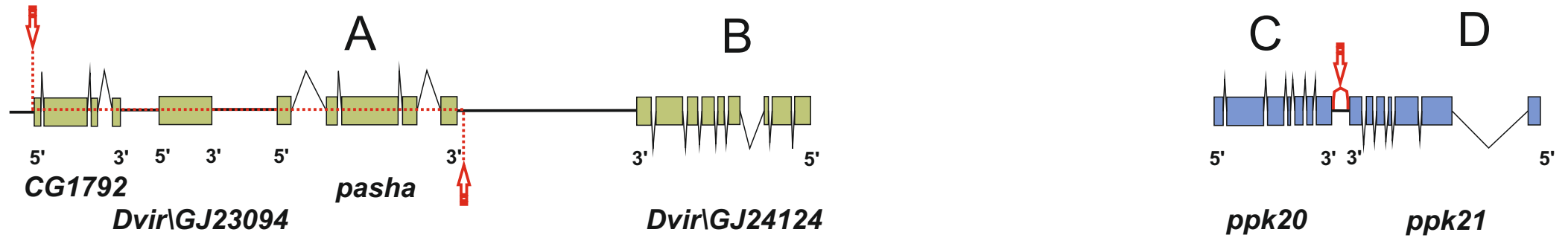

*D. mojavensis*

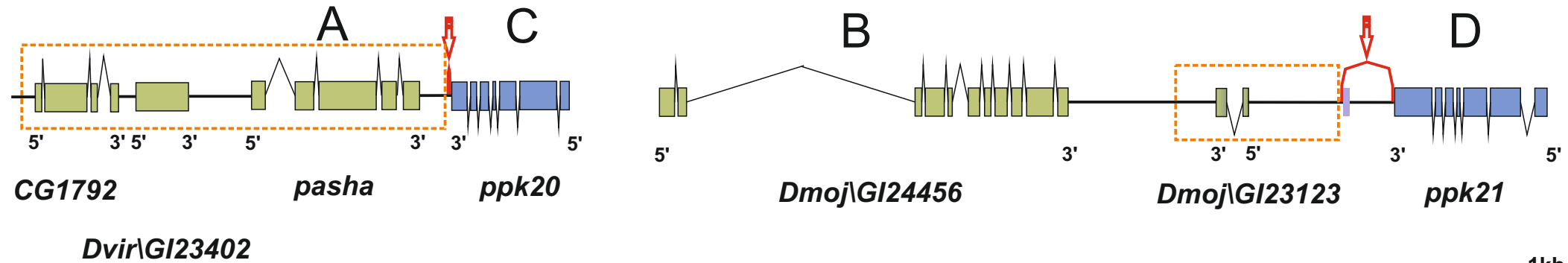

1kb

Supplement: Additional file 3 — Annotation of inversion 2h breakpoint regions. Annotation of inversion 2h distal and proximal breakpoint regions in D. virilis (non-inverted chromosome) and D. mojavensis (inverted chromosome). Inverted duplications in the D. mojavensis breakpoints are enclosed within dotted boxes, orange color. That in region AC (7.1 kb) is intact whereas that in region BD (2.7 kb) has suffered several deletions. These duplications were presumably generated by staggered single-strand breaks in the parental chromosome represented by a dotted red lines flanked by red arrows. A fragment of BuT3 is shown as a blue rectangle in region BD. Other symbols as in Figure 4. [file 1471-2164-13-53-S3.PDF]

2g

*D. virilis*

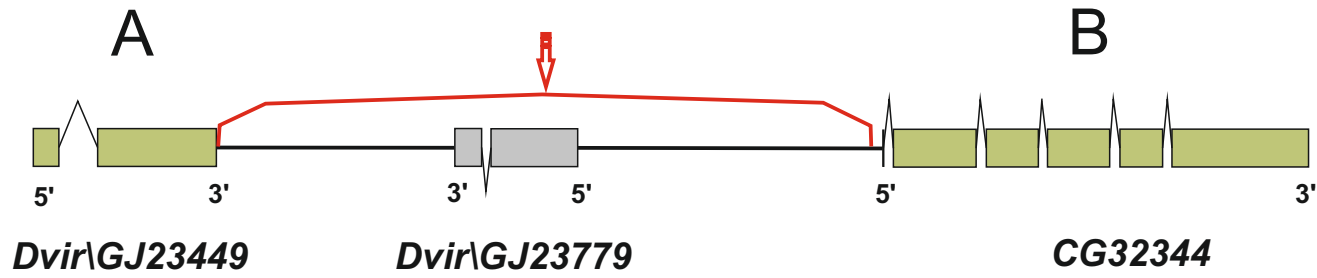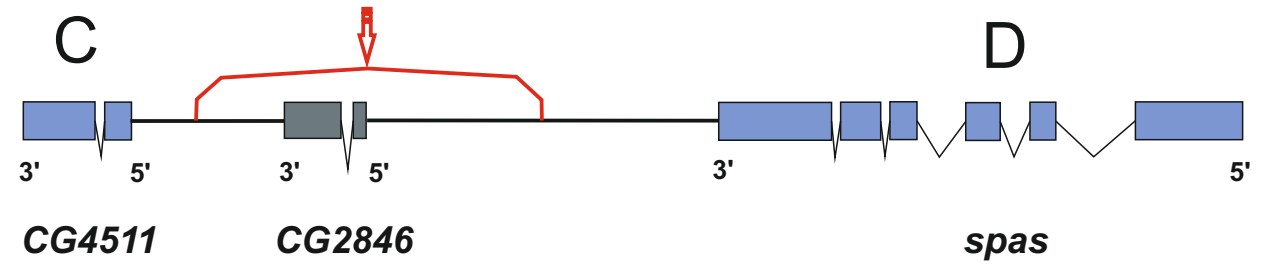

*D. mojavensis*

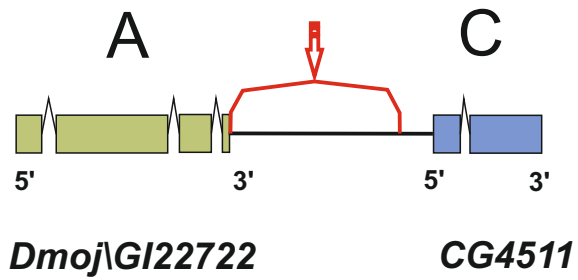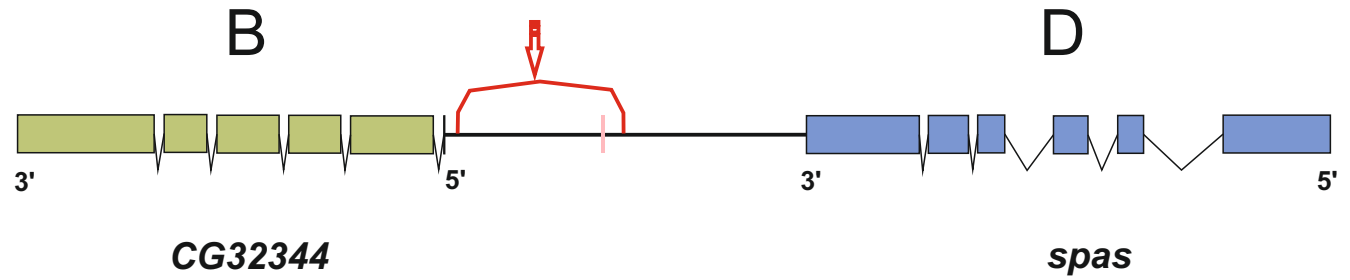

500 bp

Supplement: Additional file 4 — Annotation of inversion 2g breakpoint regions. Annotation of inversion 2g distal and proximal breakpoint regions in D. virilis (non-inverted chromosome) and D. mojavensis (inverted chromosome). Two D. virilis lineage specific genes are shown as grey rectangles. Other symbols as in Figure 4. [file 1471-2164-13-53-S4.PDF]

2f

*D. virilis*

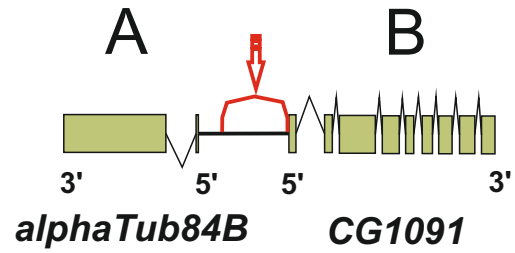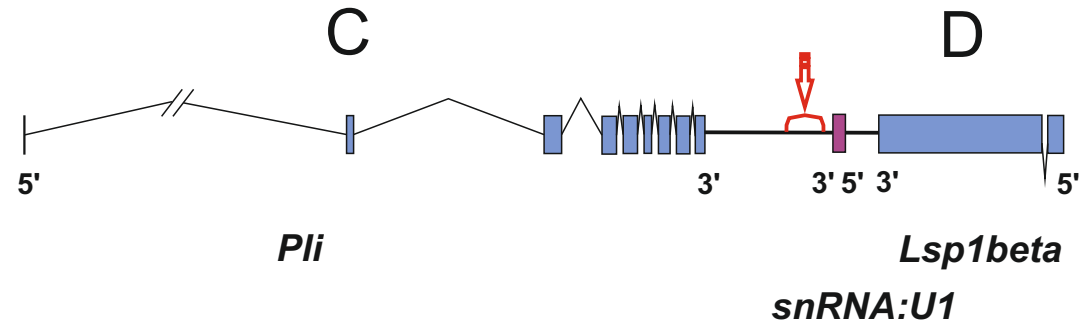

*D. mojavensis*

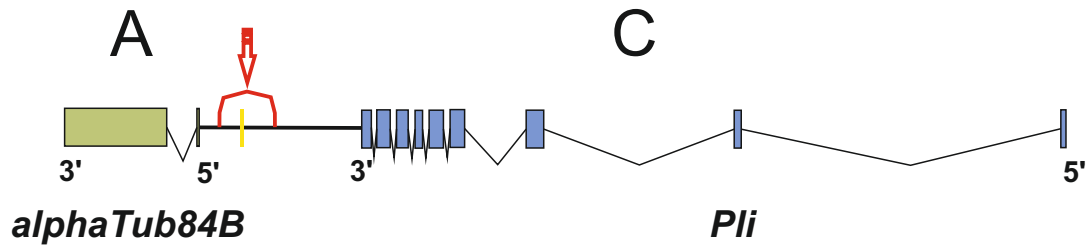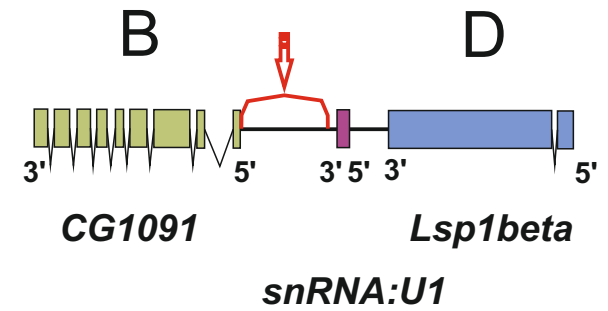

1kb

Supplement: Additional file 5 — Annotation of inversion 2f breakpoint regions. Annotation of inversion 2f distal and proximal breakpoint regions in D. virilis (non-inverted chromosome) and D. mojavensis (inverted chromosome). Symbols as in Figure 4. [file 1471-2164-13-53-S5.PDF]

*D. virilis*

2c

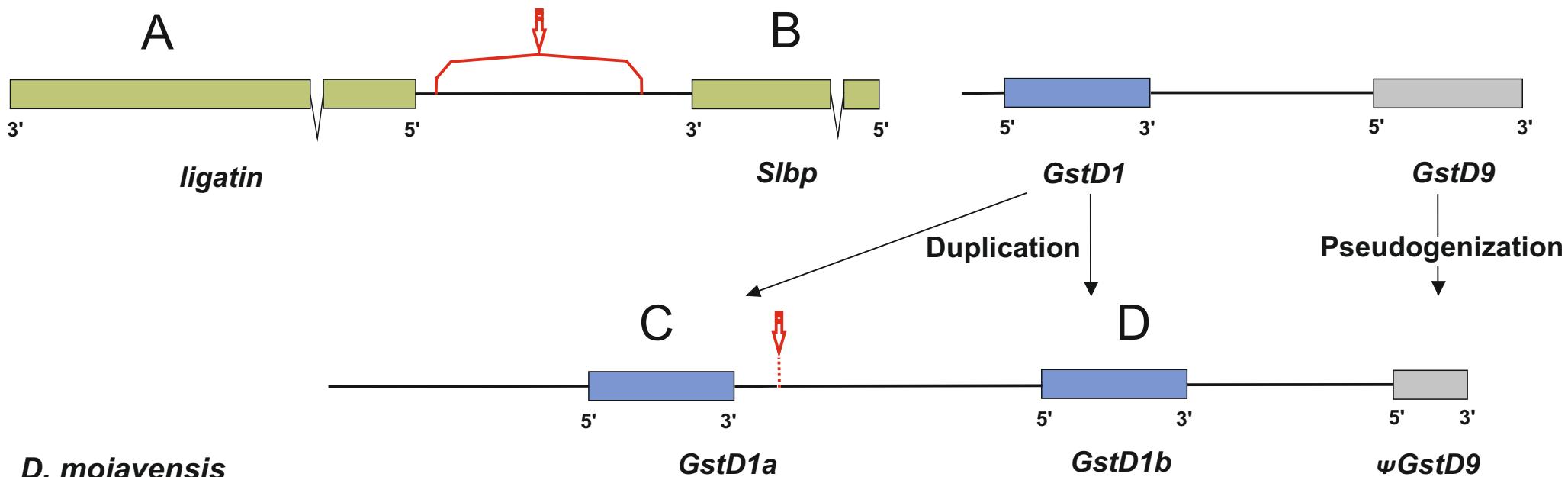

*D. mojavensis*

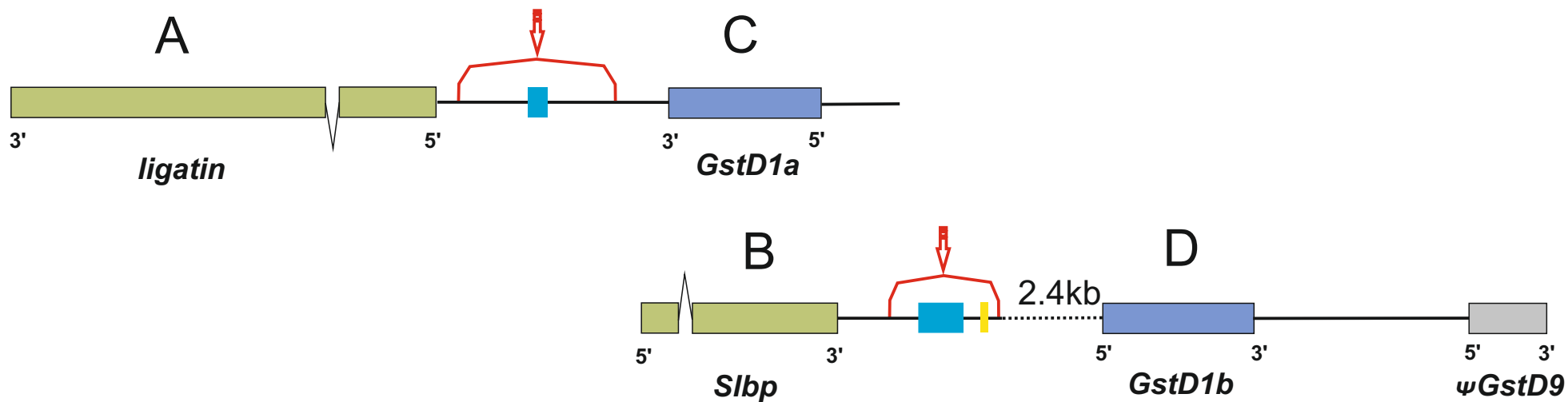

250 bp

Supplement: Additional file 6 — Annotation of inversion 2c breakpoint regions. Annotation of inversion 2c distal and proximal breakpoint regions in D. virilis (non-inverted chromosome) and D. mojavensis (inverted chromosome). Phylogenetic analysis of GstD genes (Additional file 8) indicates that the 2c inversion occurred after the duplication of the GstD1 gene in the parental chromosome. The GstD9 gene has lost its function in D. mojavensis becoming a pseudogene. Other symbols as in Figure 4. [file 1471-2164-13-53-S6.PDF]

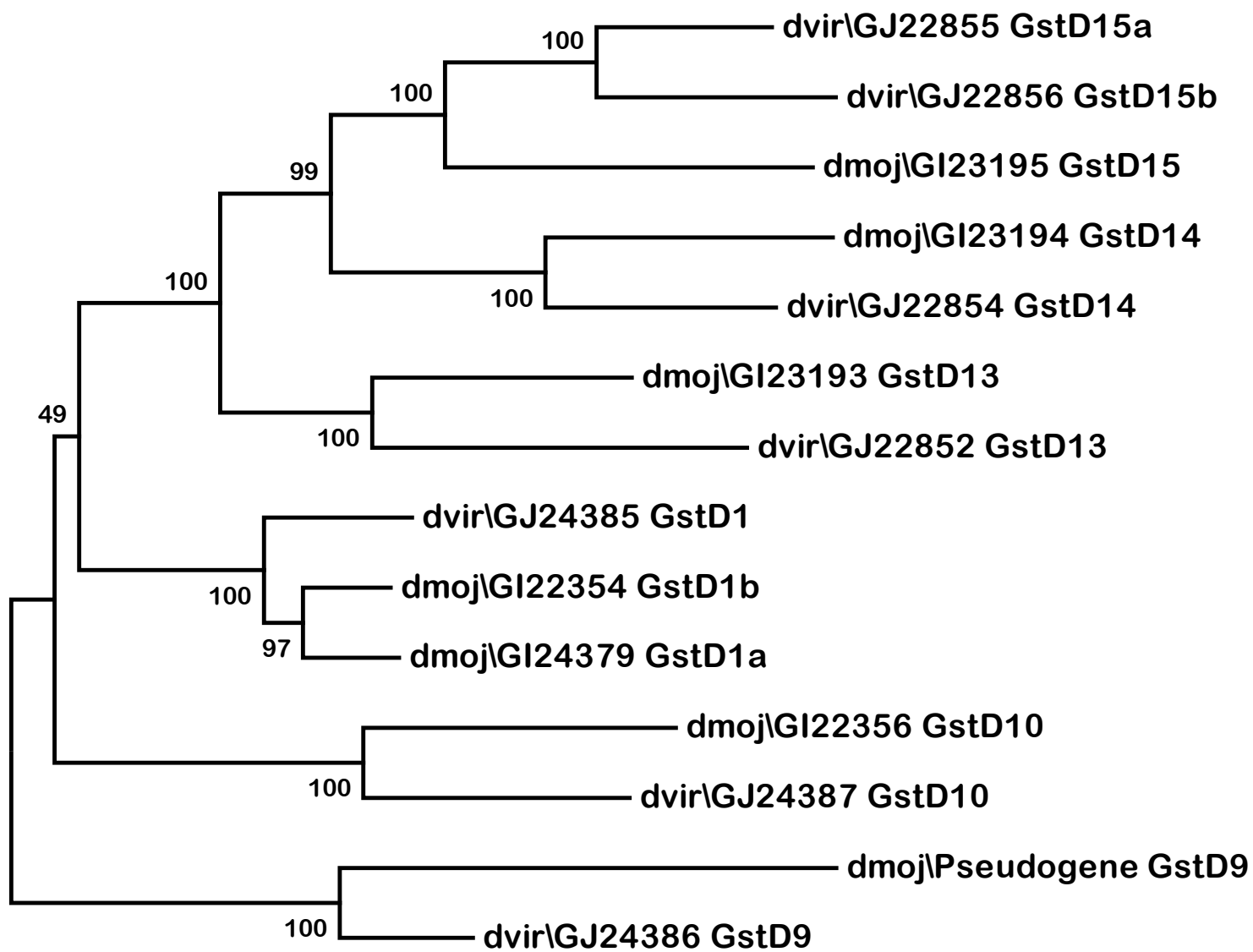

0.05

Supplement: Additional file 8 — Neighbor-Joining phylogenetic tree of GstD genes in D mojavensis and D virilis. Neighbor-Joining phylogenetic tree of GstD genes in D mojavensis and D virilis. Bootstrap values data for all tree nodes are shown. Phylogenetic analysis was conducted with MEGA4 [114]. Evolutionary distances were computed using the Maximum Composite Likelihood method. [file 1471-2164-13-53-S8.PDF]

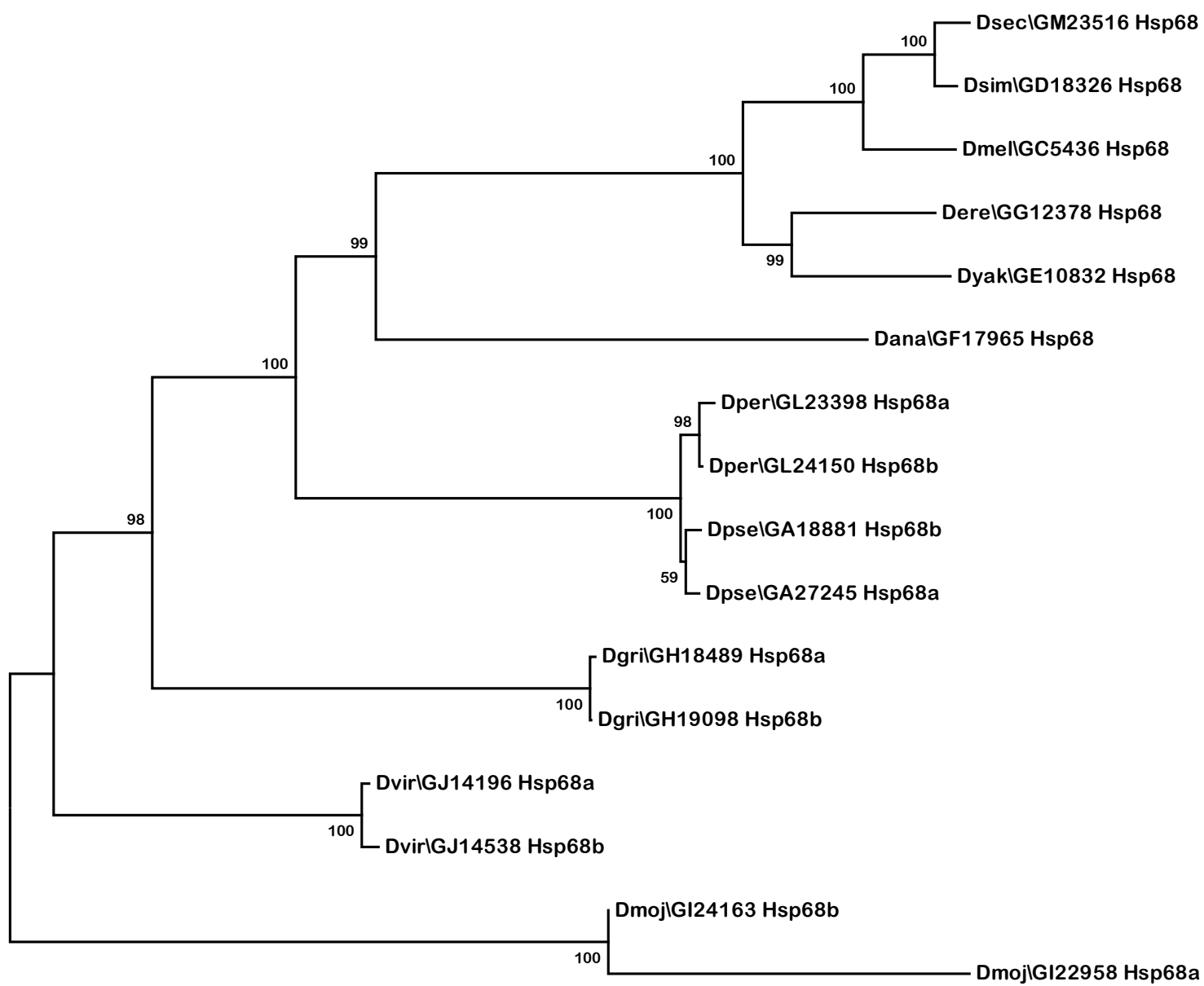

0.02

Supplement: Additional file 9 — Neighbor-Joining phylogenetic tree of Hsp68 genes of 12 sequenced Drosophila species. Neighbor-Joining phylogenetic tree of Hsp68 genes of 12 sequenced Drosophila species. D. persimilis, D. pseudoobscura, D. grimshawi, D. virilis and D. mojavensis have two copies of the Hsp68 gene, while D. sechellia, D. simulans, D. melanogaster, D. erecta, D. yakuba and D. ananassae only one. No Hsp68 gene has been detected in D. willistoni. Bootstrap values for all tree nodes are shown. Phylogenetic analysis was carried out using MEGA4 [114]. Evolutionary distances were computed using the Maximum Composite Likelihood method. [file 1471-2164-13-53-S9.PDF]

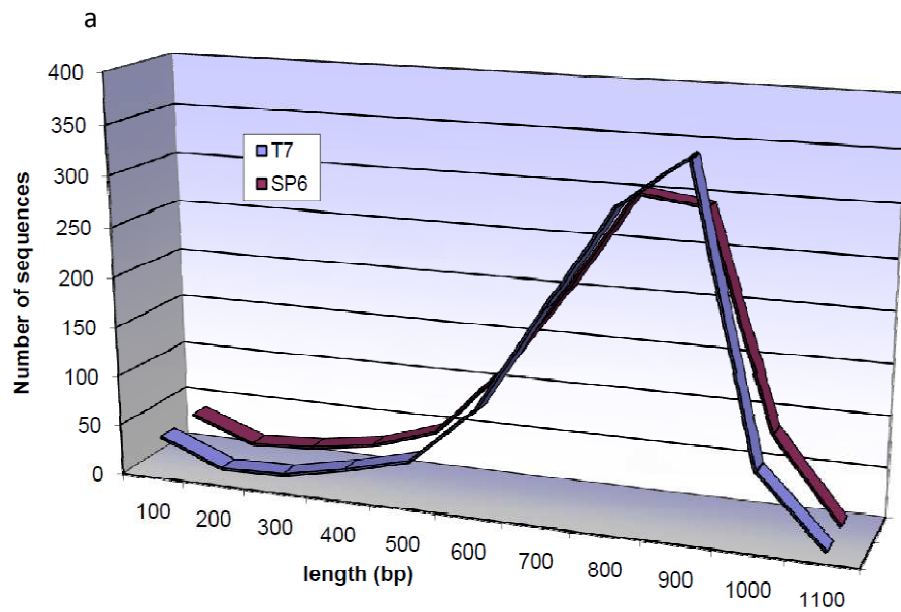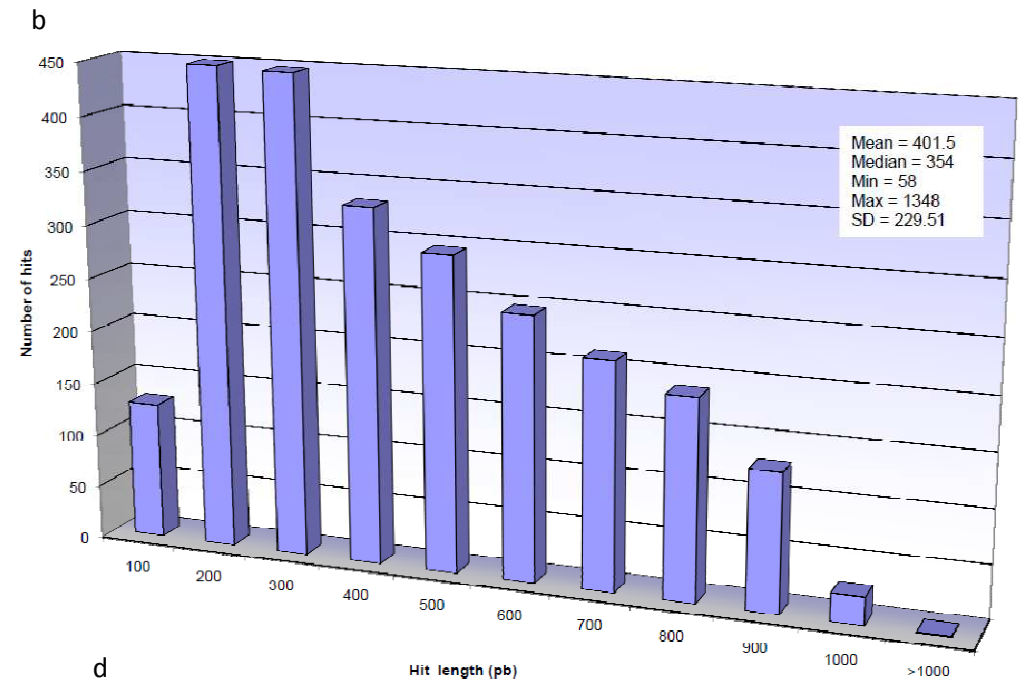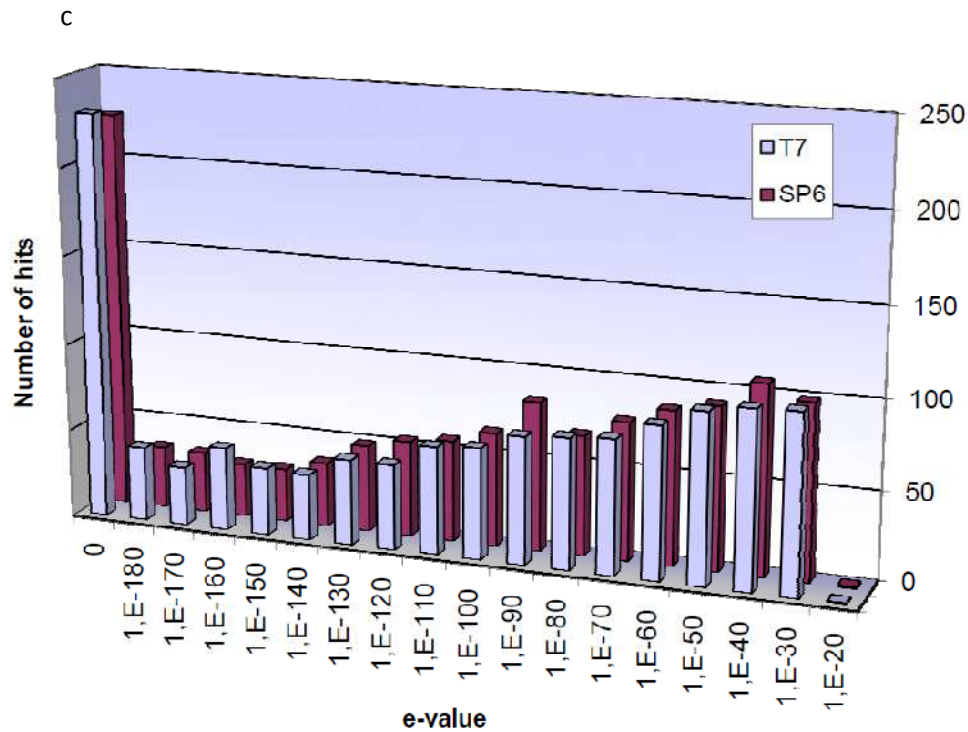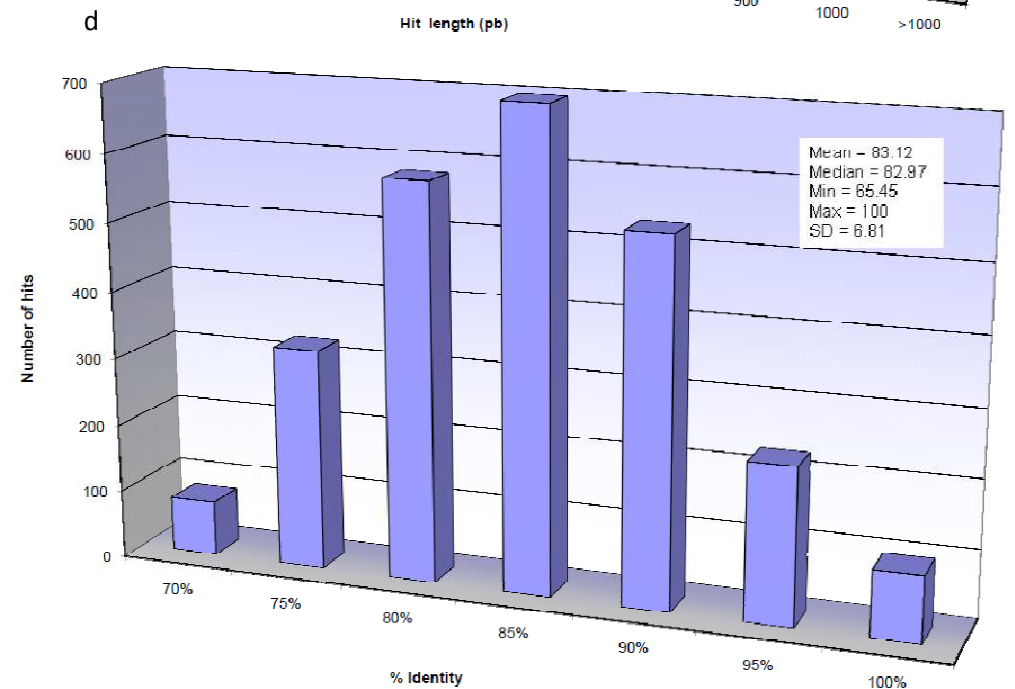

Supplement: Additional file 10 — Statistics of D. buzzatii BAC end sequences. Description: Size distribution of D. buzzatii BAC end sequences (A) and distribution of size (B), E-value (C) and % identity (D) for hits generated blasting them against the D. mojavensis genome. See text for details. [file 1471-2164-13-53-S10.PDF]
