# Supplementary material for: Gene alterations at Drosophila inversion breakpoints provide prima facie evidence for natural selection as an explanation for rapid chromosomal evolution
Source: BMC Genomics. 2012 Feb 1;13:53. doi: 10.1186/1471-2164-13-53 (PMC3355041; doi:10.1186/1471-2164-13-53)
Supplement: Additional file 7 — TE content of inversion breakpoint regions in D. mojavensis. [file 1471-2164-13-53-S7.PDF]

Additional file 7. TE content of inversion breakpoint regions in *D. mojavensis*.

| Inversion | Breakpoint | TE library | ReAS equivalence | Name | Breakpoint region coordinates | | Length | Direction | % Identity | E-value |
|---|---|---|---|---|---|---|---|---|---|---|
| | | | | | Begin | End | | | | |
| *2c* | Proximal | *BuT*5 | dmoj_292 | *BuT*5 | 117 | 175 | 59 | forward | 77 | 3.00E-003 |
| | | Repbase | | *Galileo D. willistoni* | 373 | 402 | 30 | reverse | 86,7 | 7.35E-003 |
| | Distal | *BuT*5 | dmoj_292 | *But5* | 261 | 302 | 42 | forward | 88 | 5.00E-008 |
| *2f* | Distal | RepBase | dmoj_472 | *Galileo D. buzzatii* | 359 | 409 | 51 | forward | 82,7 | 6.83E-009 |
| *2g* | proximal | RepBase | | *Transib2_DP D.pseudoobscura* | 960 | 983 | 24 | reverse | 95,8 | 2.71E-003 |
| *2h* | proximal | RepBase | dmoj_700 | *BuT*3 | 37 | 143 | 107 | forward | 100 | 5.36E-005 |
| *2q* | proximal | ReAS | dmoj_510 | ?* | 1 | 246 | 246 | reverse | 100 | 1.00E-116 |
| | | | dmoj_550 | ?* | 387 | 467 | 81 | reverse | 100 | 9.00E-07 |
| *2s* | proximal | *BuT*5 | | *BuT*5 | 224 | 250 | 27 | reverse | 100 | alignment |
| | Distal | *BuT*5 | dmoj_292 | *BuT*5 | 502 | 1482 | 981 | forward | 100 | 0 |
| | | *Galileo* | | *Galileo D. mojavensis* | 1555 | 1519 | 37 | reverse | 81,1 | 1.82E-004 |
| | | RepBase | dmoj_487 | *Homo3 hAT D.mojavensis* | 23 | 501 | 479 | forward | 93,3 | 3.29E-125 |
| *2r* | Proximal** | Repbase | dmoj_361 | *Homo6 hAT D.mojavensis* | 58 | 468 | 111 | reverse | 75,5 | 1.03E-015 |
| | | *Galileo* | dmoj_257 | *Galileo D. mojavensis* | 520 | 1308 | 789 | forward | 86 | 0 |
| | | | dmoj_472 | *Galileo D. willistoni* | 1308 | 1373 | 66 | forward | 75 | 3.06e-05 |
| | distal | nr NCBI | dmoj_257 | *Galileo D.mojavensis* | 12 | 279 | 268 | forward | 78 | 8.00E-051 |
| | | | | *Galileo D.mojavensis* | 1368 | 1430 | 63 | reverse | 83 | 9.00E-006 |
| | | RepBase | dmoj_492 | *Galileo D. willistoni* | 573 | 641 | 69 | reverse | 72.8 | 2.34E-006 |
| | | *Galileo* | dmoj_270 | *Galileo D. mojavensis* | 547 | 563 | 17 | forward | 88.2 | alignment |
| | | RepBase | | *Invader5 D.melanogaster* | 1305 | 1332 | 28 | reverse | 96,4 | 2.86E-005 |

*TE non identified but annotated as ReAS elements.

**We only show TEs localized at both ends of the breakpoint region. The region containing the histone clusters and other TEs is not annotated here due to space restriction.
